# The KANSL1-ARL17A fusion gene generates oncogenic chKANSARL and F-circKA RNAs that synergistically drive lung cancer progression *via* a novel F-circKA/miR-6860/chKANSARL axis

**DOI:** 10.1016/j.jbc.2026.111170

**Published:** 2026-01-20

**Authors:** Xinchao Guan, Tao Liu, Sili Chen, Junwei Zhang, Huanliang Huang, Dexiong Chen, Qiaoyuan Yang

**Affiliations:** 1 The Institute for Chemical Carcinogenesis, School of Public Health, Guangzhou Medical University, Guangzhou, China; 2 Guangdong Provincial Key Laboratory of Major Obstetric Diseases, Guangdong Provincial Clinical Research Center for Obstetrics and Gynecology, The Third Affiliated Hospital of Guangzhou Medical University, Guangzhou, China

**Keywords:** fusion gene, chimeric RNA, fusion circRNA, KANSL1-ARL17A, lung cancer, miRNA

## Abstract

Fusion genes are pivotal drivers of tumorigenesis, often generating oncogenic chimeric RNAs and fusion circular RNAs. However, the mechanisms by which these transcripts synergistically contribute to cancer progression remain poorly understood. Here, we identified a lung cancer-specific chimeric RNA KANSL1-ARL17A (chKANSARL) and its circular variant fusion circular RNA KANSL1-ARL17 A (F-circKA), both derived from the fusion gene KANSARL. Functional assays revealed that overexpression of either chKANSARL or F-circKA significantly enhanced lung cancer cell proliferation, migration, and invasion, while their knockdown suppressed these malignant phenotypes. *In vivo* experiments demonstrated that chKANSARL overexpression accelerated tumor growth in immunodeficient mice. Notably, coexpression experiments uncovered a synergistic regulatory interaction between F-circKA and chKANSARL, amplifying oncogenic effects. Mechanistically, miRNA sequencing and dual-luciferase assays revealed that F-circKA acts as a molecular sponge for miR-6860, thereby derepressing chKANSARL expression. Rescue experiments further validated this regulatory axis, wherein miR-6860 inhibition reversed the tumor-suppressive effects of F-circKA knockdown. Collectively, our study identifies and characterizes a novel F-circKA/miR-6860/chKANSARL regulatory axis, revealing how dual transcriptional outputs from the KANSARL fusion gene can synergistically drive lung cancer progression. These findings highlight a previously unrecognized layer of cooperative regulation between linear and circular fusion RNAs in oncogenesis and provide a new framework for understanding fusion gene-mediated tumorigenesis.

Lung cancer remains one of the most lethal malignancies worldwide, accounting for over 1.8 million deaths annually and posing a significant public health burden (1). While risk factors such as smoking, environmental carcinogens, and chronic inflammation contribute to its pathogenesis, genomic instability—a hallmark of cancer—plays a central role in driving oncogenic transformations (2, 3, 4, 5). Chromosomal rearrangements, including translocations, deletions, and inversions, frequently generate fusion genes that disrupt normal cellular regulation. These aberrations often produce chimeric RNAs (chRNAs) or fusion circular RNAs (F-circRNAs), which serve as critical drivers of tumor initiation and progression through diverse mechanisms (6, 7, 8). For example, the BCR-ABL1 fusion in chronic myeloid leukemia encodes a constitutively active tyrosine kinase, while the EML4-ALK fusion in non-small cell lung cancer (NSCLC) activates oncogenic signaling cascades (9, 10). Beyond protein-coding chRNAs, non-coding fusion transcripts such as SLC45A3-ELK4 and F-circRNAs like F-circBA1 further exemplify the multifaceted roles of fusion gene products in promoting proliferation, metastasis, and therapy resistance (11, 12). Despite these advances, the cooperative mechanisms by which linear and circular transcripts from the same fusion gene synergistically enhance malignancy remain poorly understood.

F-circRNAs, a recently discovered class of non-coding RNAs, exhibit unique biological properties due to their covalently closed structures (13, 14). Unlike linear RNAs, F-circRNAs resist exonuclease degradation, enabling prolonged cytoplasmic retention and functional persistence (15, 16). These molecules often act as miRNA sponges or protein scaffolds to amplify oncogenic pathways (17). For instance, F-circSR1 and F-circSR2, derived from the SLC34A2-ROS1 fusion in lung adenocarcinoma, enhance cell migration by sequestering tumor-suppressive miRNAs (18). Similarly, F-circEA-2a from the EML4-ALK fusion promotes NSCLC progression through ALK pathway activation (19). However, whether F-circRNAs interact with their linear counterparts to establish feedback loops or compensatory networks remains an open question (20, 21). This knowledge gap underscores the need to explore fusion gene products as integrated systems rather than isolated entities.

The KANSL1-ARL17A (KANSARL) fusion gene, initially identified in prostate cancer and glioblastoma, represents a compelling yet understudied oncogenic driver (22). Located on chromosome 17q21.31, KANSARL arises from the rearrangement of KANSL1 (a chromatin modifier) and ARL17A (a GTPase involved in vesicular trafficking) (23). Notably, KANSARL exhibits ethnic specificity, with higher prevalence in European populations, and is associated with epigenetic dysregulation and recurrent read-through fusion transcripts in cancer (24). While it’s linear chimeric RNA (chKANSARL) has been detected in multiple malignancies, including lung cancer, its functional role and potential circular RNA derivatives remain unexplored. Critically, no studies have addressed whether KANSARL generates F-circRNAs or how its transcriptional outputs coordinate to drive tumorigenesis.

In this study, we combined bioinformatics, molecular biology, and functional assays to unravel the oncogenic roles of KANSARL-derived transcripts in lung cancer. Using RNA-seq data from 13 lung cancer cell lines and two normal bronchial epithelial cell lines, we identified a lung cancer-specific chimeric RNA, chKANSARL, and validated its origin from the KANSARL fusion gene using a novel Bisection Nested PCR strategy. Furthermore, we discovered a circular RNA isoform, fusion circular RNA KANSL1-ARL17 A (F-circKA), transcribed from the same fusion locus. Through gain- and loss-of-function experiments, we demonstrated that both chKANSARL and F-circKA promote cell proliferation, migration, and invasion *in vitro* and *in vivo*. Strikingly, coexpression analyses revealed a synergistic relationship between these transcripts, with F-circKA acting as a miR-6860 sponge to stabilize chKANSARL expression. Our findings uncover a previously unrecognized regulatory axis involving F-circKA, miR-6860, and chKANSARL. We demonstrate how these dual transcriptional outputs are functionally linked, providing a novel example of cooperative oncogenic signaling originating from a single fusion gene.

## Results

### Prediction, screening and identification of chimeric RNA KANSL1-ARL17A in lung cancer

**Figure 1:**
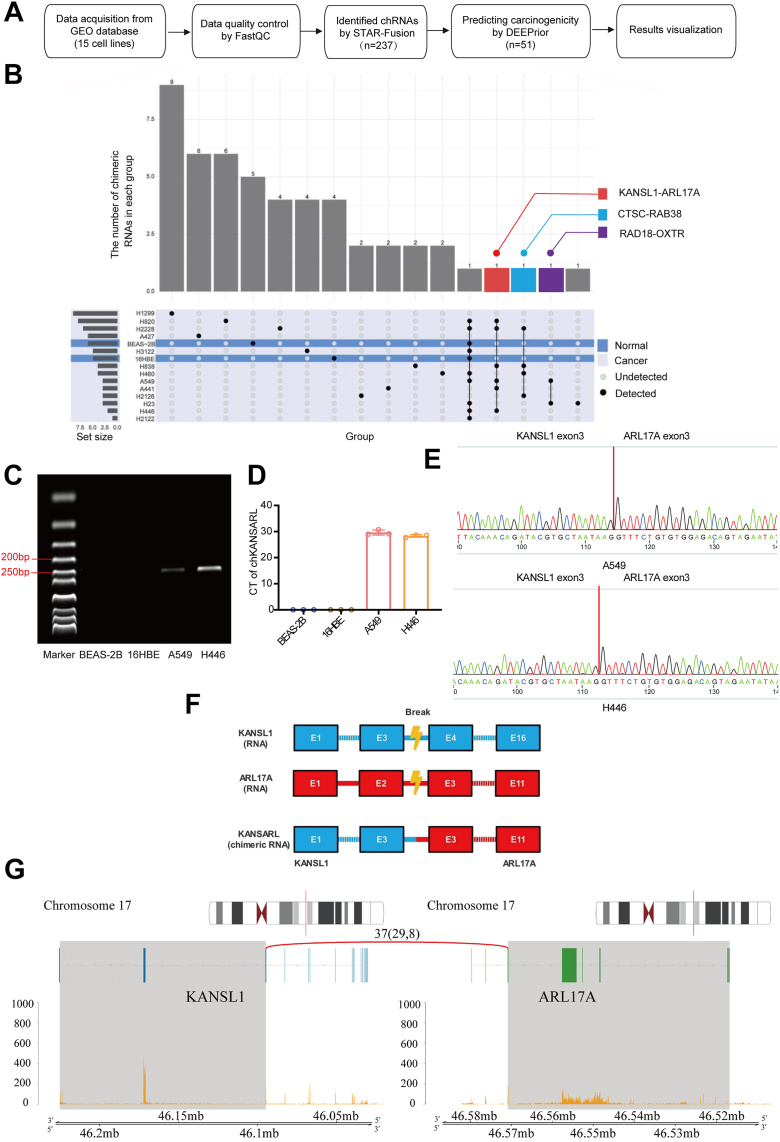
**Identification and validation of lung cancer-specific chimeric RNA chKANSARL.***A*, flow chart of chRNAs prediction. *B*, an upset plot illustrating the distribution of carcinogenic chRNAs across different cell lines. Normal lung epithelial cells and lung cancer cell lines are denoted by *blue* and *gray*, respectively. Each vertical axis represents a group, indicating the varying intersections of chRNAs across different cell lines (a *black dot* signifies prediction in a single cell line, while multiple *black dots* indicate simultaneous prediction in multiple cell lines). The *bar chart* above depicts the number of chRNAs in each group, and the set size on the *left* represents the total number of predicted chRNAs in each cell line. *C*, the expression of linear sequence chKANSARL was verified by agarose gel electrophoresis using convergent primers that span the linear fusion junction. The RT-PCR product of chKANSARL was 237 bp. *D*, the qRT-PCR results are consistent with the agarose gel electrophoresis results. Data are shown as mean ± SD from n = 3 independent experiments, with individual data points plotted. *E*, sanger sequencing confirms the RNA-level chimeric junction of chKANSARL. The *red line* indicates the fusion site within the transcript. *F*, schematic representation of chKANSARL formation. *G*, a schematic diagram showing reads spanning the fusion junction. chRNA, chimeric RNA; chKANSARL, chimeric RNA KANSL1-ARL17A.

The RNA-seq data used in this study were sourced from the GEO database. This dataset included two normal human bronchial epithelial cell lines (BEAS-2B and 16HBE) and 13 human lung cancer cell lines (A427, A549, H446, H460, H1299, H2122, H2126, H2228, H23, H3122, H441, H820, and H838). Data from 15 cell lines were obtained from the GEO database. Quality control was conducted using FastQC to ensure data reliability. chRNAs were identified through STAR-Fusion, resulting in 237 candidates. Among these, 51 chRNAs with potential carcinogenicity were further predicted using DEEPrior, and the results were visualized accordingly (Fig. 1*A*). 237 chRNAs were predicted according to the criteria, indicating its potential expression in at least one of 13 lung cancer cell lines. We found that most chRNAs were located on chromosome 17, while the fewest were on chromosome Y, which implied a possible link between chromosome 17 and the high frequency of chRNA formation in lung cancer (Fig. S1*A*). Comparative analysis revealed that the formation patterns of chRNAs, both intrachromosomal and interchromosomal, were similar between normal cell lines (Fig. S1*B*) and lung cancer cell lines (Fig. S1*C*). This similarity suggests that the basic mechanisms of chRNA formation are conserved and may play a role in both normal and cancerous cells. However, specific chRNAs that are prevalent in lung cancer cells may contribute to tumorigenesis and cancer progression. DEEPrior software (https://github.com/bioinformatics-polito/DEEPrior) was then used to predict the carcinogenicity of the remaining chRNAs. As shown in Fig. S1*D*, a total of 51 chRNAs were classified as carcinogenic (Oncogenic Probability ≥ 0.5). To focus on chRNAs that are more likely to be associated with lung cancer, we excluded chRNAs that were detected in only one lung cancer cell line or were also detectable in normal cell lines. This step ensured that the selected chRNAs were more specifically associated with lung cancer. As a result, three chRNAs were identified as potential lung cancer-associated chRNAs: KANSL1-ARL17A (detection rate of 46.15%), CTSC-RAB38 (detection rate of 30.77%), and RAD18-OXTR (detection rate of 15.38%) (Fig. 1*B*). ChRNA KANSL1-ARL17A, designated as chKANSARL, was selected for further analysis for its highest detection rate in lung cancer cell lines. Gel electrophoresis revealed specific amplification products in A549 and H446 cell lines, while no amplification products were observed in BEAS-2B and 16HBE cell lines (Fig. 1*C*). The quantitative real-time PCR (qRT-PCR) results were consistent with the agarose gel electrophoresis findings, with CT values observed only in A549 and H446 cell lines (Fig. 1*D*). Sanger sequencing of the specific amplification products in A549 and H446 cell lines confirmed that the chKANSARL fusion transcript is generated by the splicing of exon 3 of KANSL1 with exon 3 of ARL17A (Fig. 1, *E* and *F*). This finding was consistent with earlier bioinformatics predictions. To further corroborate this finding at the transcript level, we visualized the RNA-seq data, which clearly showed reads spanning the KANSL1-ARL17A fusion junction in cancer cells, providing strong evidence for the expression of the chimeric transcript (Fig. 1*G*).

### Validation of the genomic origin of chKANSARL from the KANSARL fusion gene

**Figure 2:**
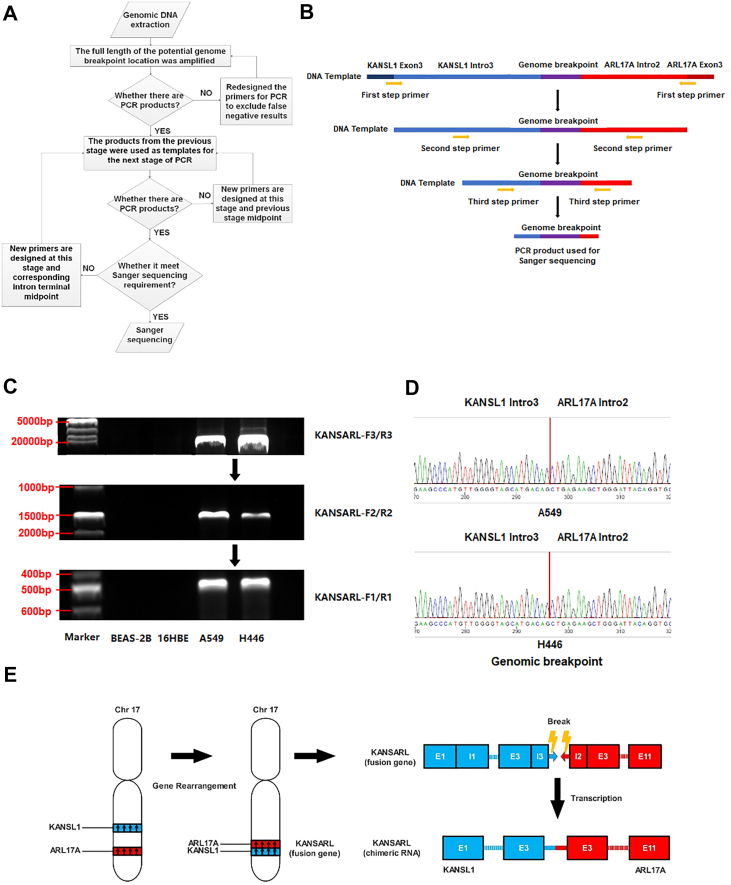
**Validating the origin of chKANSARL.***A*, schematic diagram of "Bisection Nested-PCR". *B*, approximate location map of primers used in this study. *C*, agarose gel electrophoresis of "Bisection Nested-PCR" products from lung cell lines. *D*, sanger sequencing of Bisection Nested-PCR products from A549 and H446 cells reveals the genomic breakpoint of the KANSL1-ARL17A fusion gene. The *red line* indicates the fusion site at the DNA level. *E*, schematic diagram illustrating the transcription of chKANSARL from the fusion gene KANSARL. chKANSARL, chimeric RNA KANSL1-ARL17 A.

We established and applied the “Bisection Nested-PCR” method (25, 26), as detailed in Methods section 2.4 (Genomic DNA extraction and Bisection Nested-PCR), to verify that chKANSARL originates from the fusion gene KANSARL (Fig. 2*A*). This approach allows progressive amplification of genomic regions surrounding the predicted KANSL1–ARL17A junction to determine the precise fusion site. As shown in Figure 2*B*, the stepwise PCR amplification produced progressively shorter DNA fragments as the primer pairs were moved closer to the putative junction, indicating successful enrichment of the fusion-containing region. In Figure 2*C*, the final PCR products obtained with the innermost primer pair were subjected to Sanger sequencing, which revealed a continuous sequence spanning the KANSL1 and ARL17A loci. The sequencing chromatogram (Fig. 2*D*) clearly demonstrated a genomic fusion event between intron 3 of KANSL1 and intron 2 of ARL17A, thereby validating the existence of the KANSARL fusion gene. Therefore, we infer that chKANSARL expressed in A549 and H446 cells is the transcriptional product of the fusion gene KANSARL, as shown in Figure 2*E*.

### chKANSARL promotes cells proliferation, migration, invasion and facilitates subcutaneous xenograft growth in immunodeficient mice

**Figure 3:**
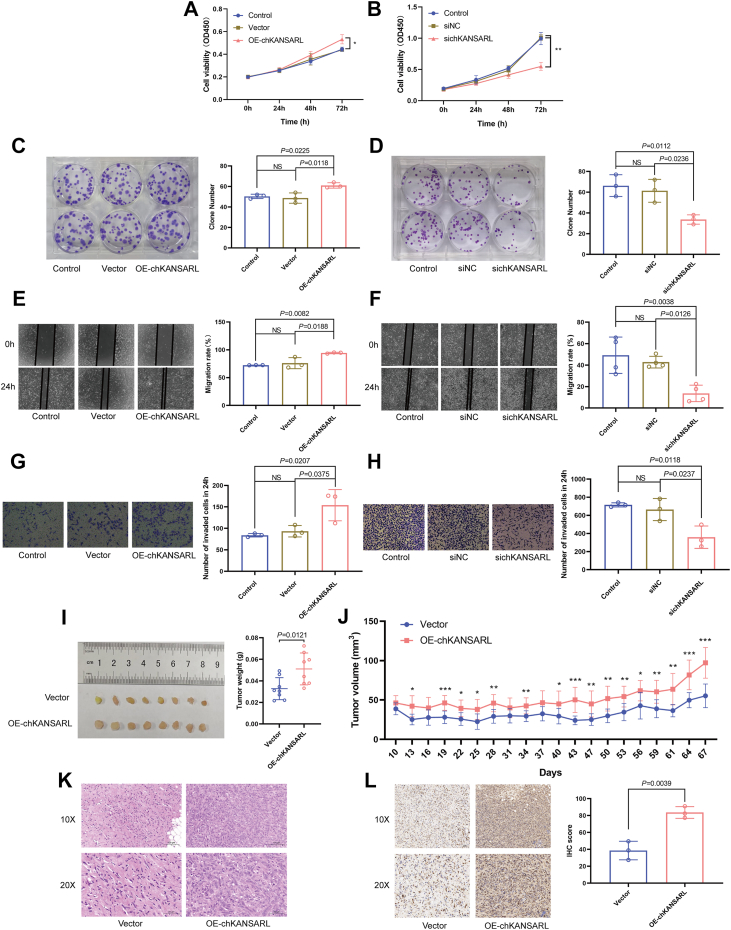
**chKANSARL enhances cells proliferation, migration, invasion, and *in vivo* tumor growth.***A* and *B*, CCK-8 assay results showing cell proliferation in chKANSARL overexpressing cells (*A*) and chKANSARL knockdown cells (*B*). *C* and *D*, plate colony formation assay results for chKANSARL overexpression (*C*) and chKANSARL knockdown (*D*). *E* and *F*, wound-healing assay results demonstrating cell migration in chKANSARL overexpressing cells (*E*) and chKANSARL knockdown cells (*F*). *G* and *H*, transwell assay results showing cell invasion in chKANSARL overexpressing cells (*G*) and chKANSARL knockdown cells (*H*). *I*, mass and (*J*) volume of subcutaneous xenografts in mice. *K*, H&E staining of tumor tissues. *L*, IHC staining for Ki-67 in tumor tissues. All data are presented as mean ± SD from n = 3 independent experiments (for *in vitro* assays) or n = 8 mice (for *in vivo* assays), with individual data points plotted. Statistical significance was determined using a two-tailed Student's *t* test or One-way ANOVA. ∗*p* < 0.05, ∗∗*p* < 0.01. chKANSARL, chimeric RNA KANSL1-ARL17A.

To validate the impact of chKANSARL on tumor proliferation, migration, and invasion, we constructed a lentiviral overexpression vector containing the chKANSARL sequence (Fig. S2*A*). Successful lentiviral transfection in BEAS-2B cells was confirmed by fluorescence microscopy (Fig. S2*B*). reverse transcription PCR (RT-PCR) and agarose gel electrophoresis verified the establishment of chKANSARL overexpressing cell lines (OE-chKANSARL) by showing specific amplification products of approximately 200 to 250 bp (Fig. S2*C*). qRT-PCR further confirmed chKANSARL overexpression in OE-chKANSARL cells (Fig. S2*D*). Western blot analysis detected only the flag protein band in both Vector and OE-chKANSARL groups, with no fusion protein band, suggesting chKANSARL functions as a non-coding RNA (Fig. S2*E*). qRT-PCR results showed chKANSARL overexpression in BEAS-2B cells with no significant impact on KANSL1 and ARL17A mRNA levels (*p* > 0.05, Fig. S2, *F* and *G*). Among the three siRNAs designed to target the chKANSARL fusion site, sichKANSARL-1 effectively suppressed chKANSARL expression in H446 cells (*p* < 0.05, Fig. S2*H*). Subsequently, sichKANSARL-1 was referred to as sichKANSARL. This siRNA had no significant impact on KANSL1 and ARL17A mRNA expression levels (*p* > 0.05, Fig. S2, *I* and *J*), indicating that chKANSARL functions independently of its parental genes, KANSL1 and ARL17A. CCK-8 and plate colony formation assays demonstrated that chKANSARL overexpression significantly enhanced cell proliferation, while its knockdown inhibited proliferation compared to controls *(p* < 0.05, Fig. 3, *A*–*D*). Wound-healing and Transwell assays further confirmed that chKANSARL overexpression promoted cell migration and invasion, whereas its knockdown suppressed these processes (*p* < 0.05, Fig. 3, *E*–*H*). *In vivo* experiments using chKANSARL-overexpressing H446 cells revealed that chKANSARL overexpression significantly promoted the growth of subcutaneous xenografts in terms of mass (Fig. 3*I*) and volume (Fig. 3*J*). Mouse body weight changes showed no significant differences (Fig. S2*K*). Histological analysis with hematoxylin-eosin staining and immunohistochemistry revealed a higher density of tumor cells and significantly elevated Ki-67 protein levels in the OE-chKANSARL group, indicating enhanced tumor cell proliferation (Fig. 3, *K* and *L*). The results above demonstrated that chKANSARL promotes cell proliferation, migration, and invasion, and enhances tumor growth *in vivo*.

### Identification and functional analysis of F-circKA, the circular transcript of the fusion gene KANSARL

**Figure 4:**
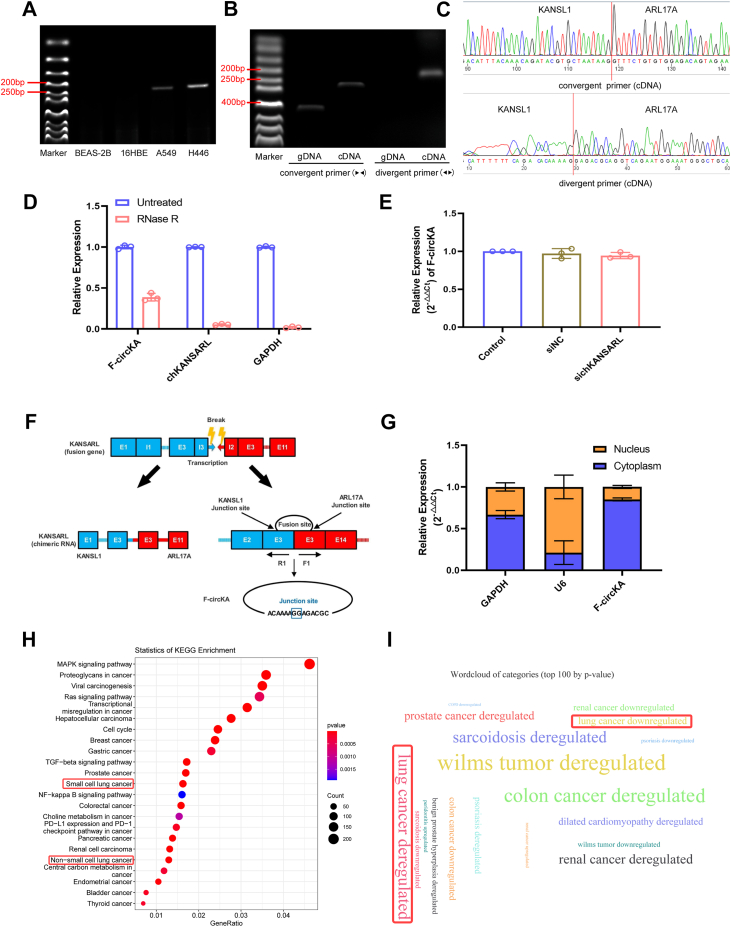
**Identification and functional prediction of F-circKA.***A*, agarose gel electrophoresis was performed on RT-PCR products from lung cell lines using divergent primers that are designed to span the backsplice junction. The RT-PCR product of circular junction F-circKA was 253 bp. *B*, convergent and divergent primers to validate F-circKA. *C*, sanger sequencing verified the sequence of F-circKA. *D*, the F-circKA expression level after RNase R treatment. Data are shown as mean ± SD from n = 3 independent experiments. *E*, qRT-PCR analyses of F-circKA expression in H446 after knockdown the expression of chKANSARL. *F*, schematic diagram of F-circKA transcription. Data are shown as mean ± SD from n = 3 independent experiments. *G*, cellular localization of F-circKA. *H*, KEGG enrichment analysis of F-circKA predicted by the miEAA tool. *I*, wordcloud of diseases of F-circKA. chKANSARL, chimeric RNA KANSL1-ARL17 A.

Recent studies have identified F-circRNAs as important transcriptional products of fusion genes, often playing critical roles in tumorigenesis (27). Building on these findings, we focused on F-circKA, a circular RNA transcript derived from the fusion gene KANSARL, to validate its existence and investigate its potential functions. Gel electrophoresis results showed amplification products of F-circKA in A549 and H446 cell lines using divergent primers specifically designed to detect the back-splice junction of circular RNAs, while no amplification products were observed in BEAS-2B and 16HBE cell lines (Fig. 4*A*). In H446 cells, convergent primers produced amplification products in both genomic DNA and complementary DNA (cDNA), whereas divergent primers produced amplification products only in cDNA, confirming the circular structure of F-circKA (Fig. 4*B*). Sanger sequencing of the products from H446 cDNA, generated by convergent and divergent primers, confirmed the complete sequence of F-circKA (Fig. 4*C*). Validation using RNase R digestion showed that F-circKA was more resistant to RNase R compared to linear mRNAs such as chKANSARL and GAPDH, suggesting its circular structure (Fig. 4*D*). Further analysis of F-circKA expression after knocking down chKANSARL in the H446 cell line revealed that F-circKA levels remained unaffected, which indicates that KANSARL independently transcribes both chKANSARL and F-circKA (Fig. 4, *E* and *F*). To determine the localization of F-circKA, we isolated nuclear and cytoplasmic RNA from H446 cells, using U6 as the nuclear reference gene and GAPDH as the cytoplasmic reference gene. F-circKA expression was found to be similar to that of GAPDH, indicating that this fusion circular RNA primarily exists in the cytoplasm (Fig. 4*G*). Based on its cytoplasmic localization and miRNA binding predictions, F-circKA represents a candidate for further investigation as a potential regulator of downstream mRNAs through miRNA interactions. We then predicted a total of 89 miRNAs that F-circKA might bind using miRanda (28) (Table S6). Disease association analysis of these miRNAs using miEAA (29) revealed enrichment in various cancer-related pathways, including NSCLC and small cell lung cancer (Fig. 4*H*), suggesting a potential role for a F-circKA/miRNA pathway in lung cancer. Additionally, miEAA analysis indicated that F-circKA might be involved in various human tumors and deregulated in lung cancer (Fig. 4*I*). Therefore, it is reasonable to suspect that F-circKA/miRNA pathway could play a important role in the occurrence and development of lung cancer.

### F-circKA promotes cells proliferation, migration and invasion

We constructed a lentiviral overexpression vector containing the F-circKA sequence for transfection into BEAS-2B cells (Fig. S3*A*). The transfection efficiency in F-circKA overexpressing cell lines (OE-F-circKA) was confirmed by fluorescence microscopy (Fig. S3*B*). RT-PCR and agarose gel electrophoresis verified the successful construction of OE-F-circKA cell lines by showing specific amplification products (Fig. S3*C*). In the qRT-PCR experiment, CT values were detected in F-circKA overexpressing cell lines, confirming the successful establishment of OE-F-circKA (Fig. S3*D*). Meanwhile, wedetermined that F-circKA overexpression had ed no significant impact on KANSL1 and ARL17A mRNA expression in BEAS-2B cells (*p* > 0.05, Fig. S3, *E* and *F*).

**Figure 5:**
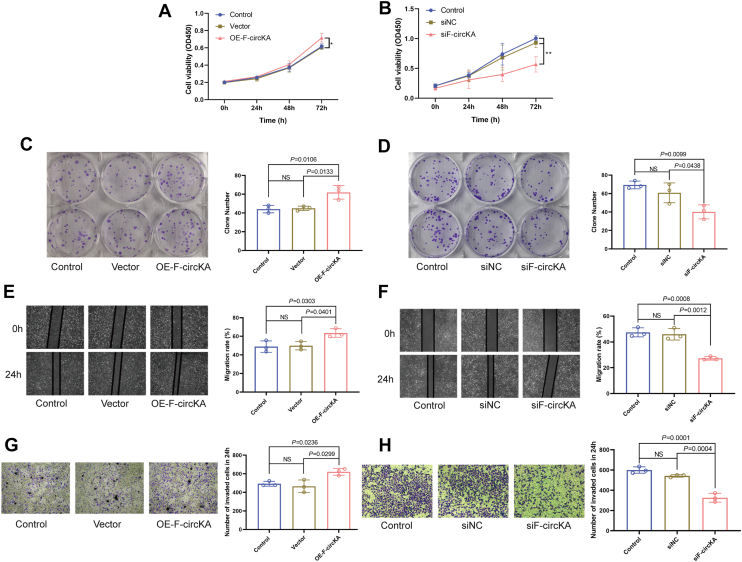
**F-circKA enhances cell proliferation, migration, and invasion.***A* and *B*, CCK-8 assay results of overexpressing the expression of F-circKA (*A*) and knocking down F-circKA (*B*). *C* and *D*, plate colony formation assay results of overexpressing the expression of F-circKA (*C*) and knocking down F-circKA (*D*). *E* and *F*, wound-healing assay results of overexpressing the expression of F-circKA (*E*) and knocking down F-circKA (*F*). *G* and *H*, transwell assay results of overexpressing the expression of F-circKA (*G*) and knocking down F-circKA (*H*). All data are presented as mean ± SD from n = 3 independent experiments, with individual data points plotted. Statistical significance was determined by a two-tailed Student's *t* test or One-way ANOVA. *p*-values are indicated on the graphs. NS, not significant. ∗*p* < 0.05, ∗∗*p* < 0.01.

Next, we designed siRNA targeting the reverse splicing site of F-circKA to knock down its expression in H446 cells. siF-circKA-3 significantly suppressed F-circKA expressionand was referred to as siF-circKA (*p* < 0.05, Fig. S3*G*). siF-circKA had no significant impact on the expression of KANSL1 and ARL17A mRNA (*p* > 0.05, Fig. S3, *H* and *I*). CCK-8 assays showed increased cell viability in the OE-F-circKA group and decreased viability in the siF-circKA group compared to their respective controls (*p* < 0.05, Fig. 5, *A* and *B*). Plate colony formation assays indicated more colonies in the OE-F-circKA group and fewer colonies in the siF-circKA group (*p* < 0.05, Fig. 5, *C* and *D*). Wound-healing assays revealed enhanced migration in OE-F-circKA cells and reduced migration in siF-circKA cells (*p* < 0.05, Fig. 5, *E* and *F*). Similarly, Transwell assays showed higher invasion ability in the OE-F-circKA group and lower invasion in the siF-circKA group compared to controls (*p* < 0.05, Fig. 5, *G* and *H*). These findings demonstrate that F-circKA promotes cell proliferation, migration, and invasion, similar to chKANSARL.

### F-circKA regulates chKANSARL expression and jointly promotes cell proliferation, migration, and invasion

**Figure 6:**
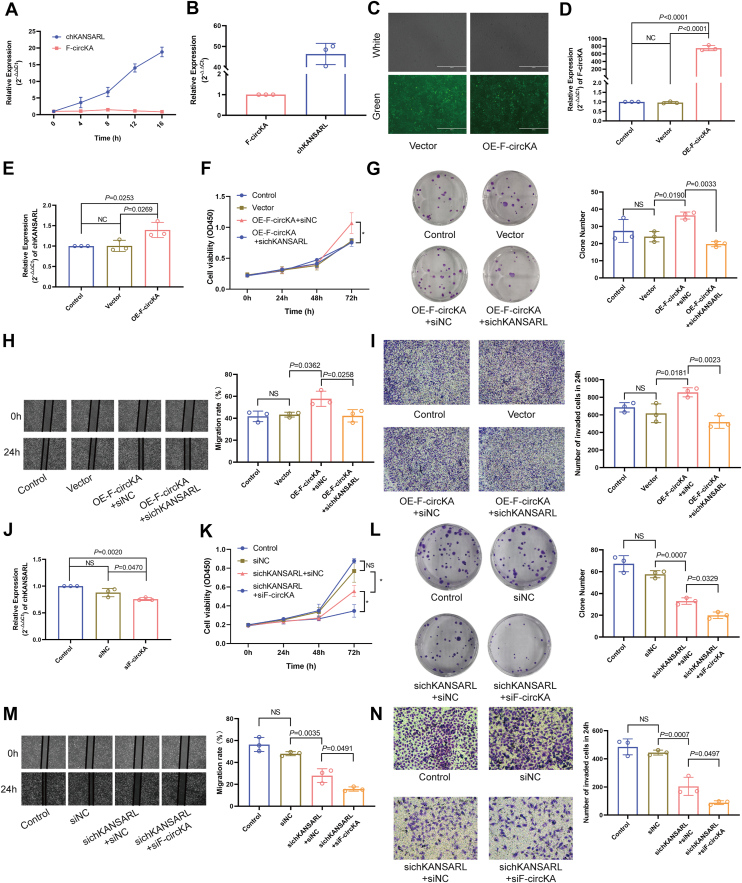
**F-circKA and chKANSARL synergistically promote malignant phenotypes.***A*, temporal expression changes of chKANSARL and F-circKA in H446 cells after passage. *B*, relative expression levels of chKANSARL and F-circKA in parental H446 cells. *C*, observation of lentivirus transfection effect by fluorescence microscope. *D*, qRT-PCR analyses confirmed the overexpression of F-circKA in H446 cells. *E*, qRT-PCR analyses of chKANSARL expression in H446 after overexpressing the expression of F-circKA. *F*, the CCK-8 assays result of knocking down chKANSARL in OE-F-circKA. *G*, the plate colony formation assays result of knocking down chKANSARL in OE-F-circKA. *H*, the wound-healing assays results of knocking down chKANSARL in OE-F-circKA. *I*, the Transwell assays results of knocking down chKANSARL in OE-F-circKA. *J*, qRT-PCR analyses of chKANSARL expression in H446 after knocking down F-circKA. *K*, the CCK-8 assays result of cotransfecting sichKANSARL and siF-circKA in H446 cells. *L*, the plate colony formation assays result of cotransfecting sichKANSARL and siF-circKA in H446 cells. *M*, the wound-healing assays results of cotransfecting sichKANSARL and siF-circKA in H446 cells. *N*, the Transwell assays results of cotransfecting sichKANSARL and siF-circKA in H446 cells. All data are presented as mean ± SD from n = 3 independent experiments, with individual data points plotted. Statistical significance was determined by a two-tailed Student's *t* test or One-way ANOVA. *p*-values are indicated on the graphs. NS, not significant. ∗*p* < 0.05. chKANSARL, chimeric RNA KANSL1-ARL17A.

To explore the probably crosstalk of the two transcription products of the fusion gene KANSARL, we observed the temporal changes in the expression levels of chKANSARL and F-circKA. a rapid increase in chKANSARL expression levels concurrent with a decrease in F-circKA expression levels was observed (Fig. 6*A*). Further analysis revealed that in parental H446 cells, the expression of chKANSARL was approximately 45-fold higher than that of F-circKA (Fig. 6*B*). Based on these observations, we speculate that a regulatory mechanism involving interactions may exist between F-circKA and chKANSARL. To investigate this, we generated an OE-F-circKA cell line using H446 cells, which naturally express chKANSARL. Fluorescence microscopy and qRT-PCR confirmed the successful establishment of the OE-F-circKA overexpression in H446 cells (Fig. 6, *C* and *D*). Overexpression of F-circKA in H446 cells led to a ∼1.3-fold increase in chKANSARL expression levels (Fig. 6*E*).

The OE-F-circKA + siNC group exhibited increased cell viability, colony formation, migration, and invasion compared to the Control and Vector groups, as shown by the CCK-8 assay (Fig. 6*F*), plate colony formation assay (Fig. 6*G*), wound healing assay (Fig. 6*H*), and Transwell assay (Fig. 6*I*). These enhancements were reversed in the OE-F-circKA + sichKANSARL group (*p* < 0.05). Knocking down F-circKA significantly reduced chKANSARL expression levels (*p* < 0.05, Fig. 6*J*). In cotransfection experiments, the siKANSARL + siNC group showed a weakened malignant phenotype, with further reductions in cell viability (Fig. 6*K*), colony formation (Fig. 6*L*), migration (Fig. 6*M*), and invasion (Fig. 6*N*) in the siKANSARL + siF-circKA group (*p* < 0.05). These results preliminarily suggest that F-circKA may be involved in the modulation of malignant phenotypes at the cell phenotype level, and there is a potential association between F-circKA and chKANSARL expression in this process.

### F-circKA modulates chKANSARL expression *via* miR-6860

**Figure 7:**
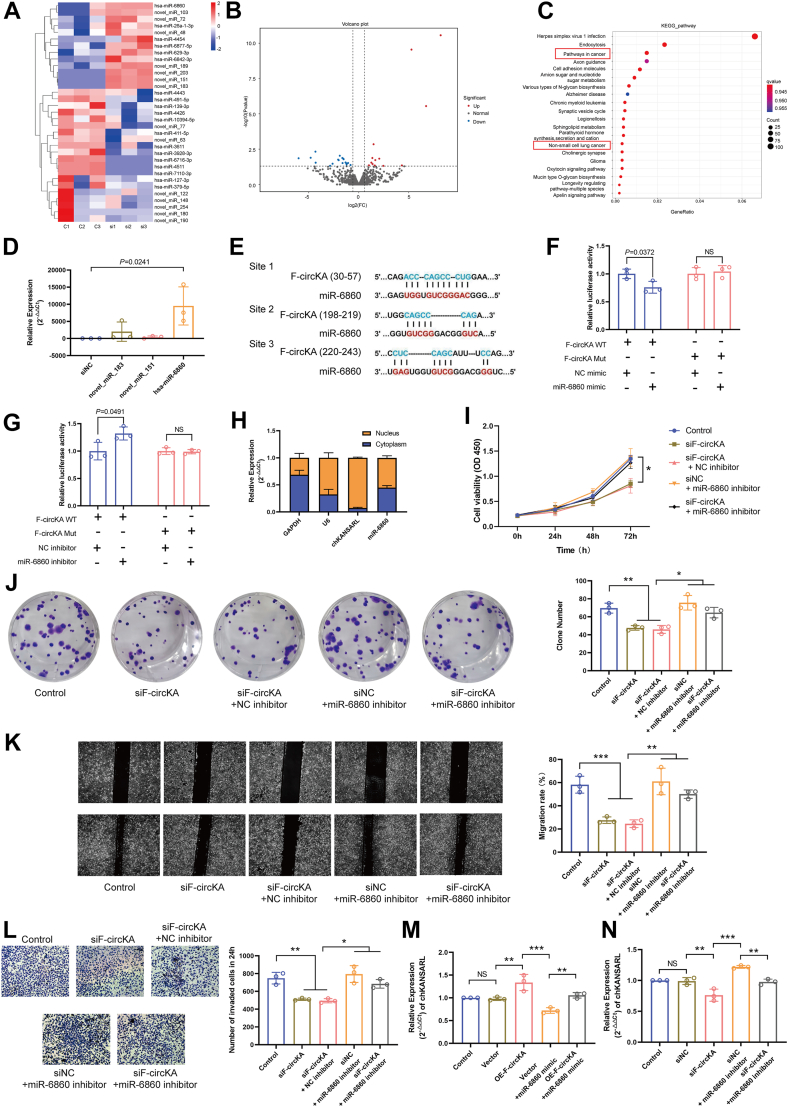
**F-circKA regulates the expression of chKANSARL through miR-6860.***A*, heatmap of differentially expressed miRNAs from RNA-seq of H446 cells after F-circKA knockdown. The color scale represents z-scores of normalized miRNA expression levels, illustrating relative upregulation (*red*) or downregulation (*blue*) for each miRNA across samples. *B*, volcano plot of differentially expressed miRNAs from RNA-seq of H446 cells after F-circKA knockdown. *C*, KEGG enrichment analysis of target genes of differentially expressed miRNAs. *D*, validation of the top three upregulated miRNAs by qRT-PCR. *E*, schematic diagram of the dual-luciferase reporter constructs. The WT (F-circKA-WT) construct contains the F-circKA sequence encompassing the three predicted miR-6860 binding sites (Site 1, 2, 3) cloned downstream of the luciferase gene. The mutant constructs (F-circKA-Mut1, Mut2, Mut3) carry specific nucleotide substitutions (detailed in Table S5) within the seed regions of the respective binding sites to abrogate miR-6860 binding. *F* and *G*, dual luciferase reporter gene assays were conducted to validate the binding relationship between F-circKA and miR-6860. *H*, examining the cellular distribution of chKANSARL and miRNA-6860. *I*, the CCK-8 assays were conducted to assess the proliferation ability of H446 cells after cotransfection with F-circKA siRNA and miR-6860 inhibitor. *J*, colony formation assays were conducted to evaluate the proliferation ability of H446 cells after cotransfection with F-circKA siRNA and miR-6860 inhibitor. *K*, wound healing assays were performed to assess the migration ability of H446 cells after cotransfection with F-circKA siRNA and miR-6860 inhibitor. *L*, transwell assays were carried out to evaluate the invasion ability of H446 cells after cotransfection with F-circKA siRNA and miR-6860 inhibitor. qRT-PCR validation of the effects of (*M*) miR-6860 mimic and (*N*) miR-6860 inhibitor on chKANSARL expression. All data are presented as mean ± SD from n = 3 independent experiments, with individual data points plotted. Statistical significance determined by a two-tailed Student's *t* test or One-way ANOVA. *p*-values are indicated. NS, not significant. ∗*p* < 0.05, ∗∗*p* < 0.01, ∗∗∗*p* < 0.001. chKANSARL, chimeric RNA KANSL1-ARL17A.

To explore the molecular mechanism of F-circKA in regulating chKANSARL, miRNA sequencing analysis was performed after downregulating F-circKA expression in H446 cells. Results showed 13 upregulated miRNAs and 20 downregulated miRNAs following F-circKA downregulation (fold changes ≥ 1.5 and *p* < 0.05) (Fig. 7, *A* and *B* and Table S7). Additionally, partial sequencing results of known miRNAs are presented in Table S8. Kyoto Encyclopedia of Genes and Genomes (KEGG) pathway analysis of the target genes of differentially expressed miRNAs identified significant enrichment in pathways related to lung cancer (Fig. 7*C*). Following the downregulation of F-circKA expression in H446 cells, qRT-PCR validation of the top three upregulated miRNAs, novel_miR_183, novel_miR_151, and miR-6860, showed that miR-6860 had the most significant upregulation. Therefore, miR-6860 was selected for further investigation (Fig. 7*D*). Using RNAhybrid (https://bibiserv.cebitec.uni-bielefeld.de/rnahybrid/), three potential binding sites between F-circKA and miR-6860 were predicted (Fig. 7*E*). F-circKA Mut sequences were designed for these three binding sites. The experimental results of dual-luciferase reporter gene assay revealed a significant decrease in relative luciferase activity upon cotransfection of the F-circKA WT and miR-6860 mimic (Fig. 7*F*). Conversely, cotransfection of the F-circKA WT and miR-6860 inhibitor led to a notable increase in relative luciferase activity, while no significant change observed when cotransfected with F-circKA Mut (Fig. 7*G*). These findings indicated a binding interaction between F-circKA and miR-6860, suggesting the functional role of F-circKA as a miRNA sponge.

In order to better understand the potential functional relationship between chKANSARL and miR-6860, we performed nuclear-cytoplasmic fractionation experiments to assess their subcellular localization. This analysis revealed that chKANSARL was predominantly localized in the cell nucleus, suggesting its involvement in nuclear non-coding RNA functions. In contrast, miR-6860 was detected in both the nucleus and cytoplasm, indicating a broader cellular distribution. These findings imply a potential interaction between miR-6860 and chKANSARL, with miR-6860 potentially modulating chKANSARL's function through its presence in different cellular compartments (Fig. 7*H*). The CCK-8 assay showed that the optical density values of H446 cells cotransfected with siF-circKA and miR-6860 inhibitor were significantly higher than the siF-circKA + NC inhibitor group (Fig. 7*I*). Similarly, the colony formation assay, wound-healing assay, and Transwell assay revealed that colony formation, migration, and invasion were enhanced in the cotransfected group compared to the siF-circKA + NC inhibitor group (Fig. 7, *J*–*L*). The qRT-PCR results revealed that transfection of miR-6860 mimic into H446 cells overexpressing F-circKA led to the restoration of chKASARL expression levels (Fig. 7*M*). Conversely, transfection of miR-6860 inhibitor into H446 cells with knocked-down F-circKA resulted in the upregulation of chKASARL expression levels (Fig. 7*N*). In summary, these results confirm that F-circKA regulates chKANSARL by sequestering miR-6860. In summary, these results confirm that F-circKA regulates chKANSARL by sequestering miR-6860, thereby modulating the cellular processes of proliferation, migration, and invasion in lung cancer cells.

## Discussion

The discovery of fusion gene-derived transcripts, including chimeric RNAs (chRNAs) and fusion circular RNAs (F-circRNAs), has revolutionized our understanding of cancer biology (30). In this study, we identified two oncogenic transcriptional products of the KANSARL fusion gene—chKANSARL and F-circKA—and elucidated their cooperative roles in driving lung cancer progression through a novel F-circKA/miR-6860/chKANSARL axis. Our findings not only expand the functional repertoire of fusion gene products but also highlight the complexity of their regulatory networks in tumorigenesis.

**Figure 8:**
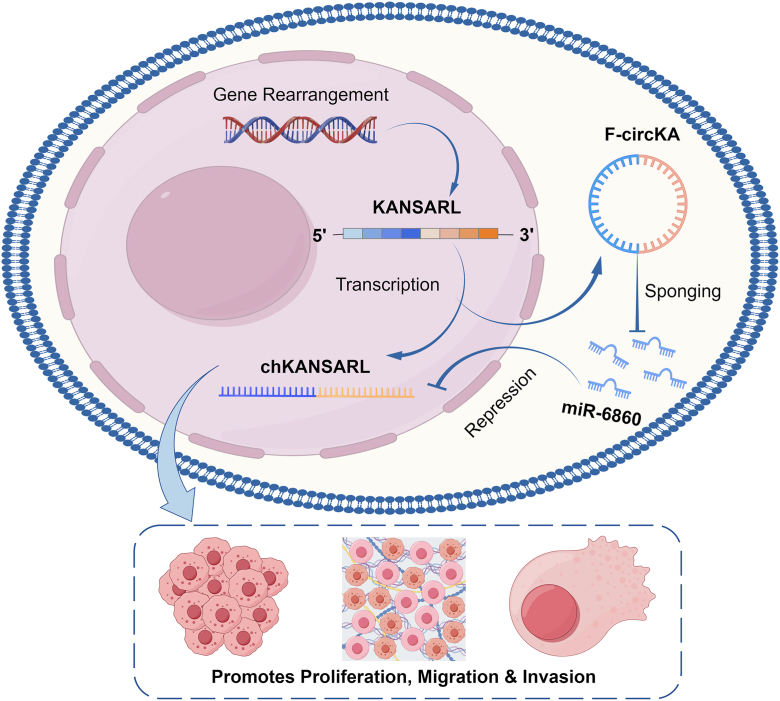
**A proposed model for the KANSARL fusion gene-driven oncogenic axis in lung cancer.** The model illustrates a multi-layered regulatory mechanism initiated by a genomic rearrangement, which forms the KANSARL fusion gene. This gene produces two distinct oncogenic transcripts: the linear chimeric RNA chKANSARL and the circular RNA F-circKA. chKANSARL is predominantly localized in the nucleus and promotes tumorigenesis. F-circKA is exported to the cytoplasm, where it functions as a competing endogenous RNA. It sequesters miR-6860, thereby relieving the miRNA-mediated repression of chKANSARL. This synergistic axis ultimately drives lung cancer cell proliferation, migration, and invasion. By Fig draw. chKANSARL, chimeric RNA KANSL1-ARL17A.

To visually summarize our findings, we propose a working model for the KANSARL-driven oncogenic axis (Fig. 8). This model illustrates the entire process, beginning from the genomic rearrangement that creates the KANSARL fusion gene. From this single locus, two distinct oncogenic transcripts are produced: the predominantly nuclear chKANSARL and the cytoplasmic F-circKA. In the cytoplasm, F-circKA functions as a competing endogenous RNA (ceRNA) by sequestering miR-6860, thereby relieving the repressive effect of this miRNA on chKANSARL. The resulting upregulation of chKANSARL, likely acting in concert with F-circKA's own functions, ultimately promotes lung cancer cell proliferation, migration, and invasion.

Both chKANSARL and F-circKA demonstrated robust oncogenic activities *in vitro* and *in vivo*. Overexpression of either transcript enhanced cell proliferation, migration, and invasion, while their knockdown suppressed these phenotypes. Notably, chKANSARL exhibited nuclear localization, suggesting potential roles in transcriptional or epigenetic regulation, whereas F-circKA predominantly localized to the cytoplasm, implicating it in post-transcriptional processes. This spatial segregation hints at a division of labor between the two transcripts, with chKANSARL potentially modulating nuclear pathways (*e.g.*, chromatin remodeling *via* KANSL1-derived domains) and F-circKA acting as a cytoplasmic effector (31). The synergistic amplification of malignant phenotypes upon coexpression further underscores their complementary functions, a phenomenon previously observed in fusion gene systems such as BCR-ABL1 and its circular isoforms (32). However, we have demonstrated that the KANSARL fusion gene produces both a linear (chKANSARL) and a circular RNA (F-circKA), and that we propose a potential regulatory model in which F-circKA may fine-tune chKANSARL expression *via* sponging miR-6860.

A key breakthrough of this study lies in deciphering the molecular crosstalk between F-circKA and chKANSARL. Through miRNA sequencing and functional validation, we identified miR-6860 as a critical mediator bridging these transcripts. F-circKA functionally interacts with miR-6860 *via* three complementary sites, sequestering this tumor-suppressive miRNA and thereby relieving its repression of chKANSARL. This mechanism aligns with the emerging paradigm of competing endogenous RNA networks, where circular RNAs stabilize target mRNAs by sponging shared miRNAs (33). Intriguingly, miR-6860 has been implicated in suppressing proliferation in gastric cancer, yet its role in lung cancer remains unexplored (34, 35). Our rescue experiments conclusively demonstrated that miR-6860 inhibition reverses the anti-tumor effects of F-circKA knockdown, solidifying its position as a central node in this regulatory axis. These findings add to the growing evidence that F-circRNAs can act as master regulators of oncogenic signaling, extending beyond their canonical roles as passive byproducts of fusion genes.

The development of the Bisection Nested PCR technique represents a significant technical advancement for fusion gene research. Traditional methods such as Fluorescence In Situ Hybridization or whole-genome sequencing, while powerful, are often cost-prohibitive or lack resolution for detecting rare fusion events (36, 37, 38, 39). By iteratively narrowing the genomic region around the fusion junction through successive PCR rounds, our approach enables rapid, high-specificity validation of chRNA origins with minimal false positives. This method proved indispensable for confirming the KANSARL genomic rearrangement and could be broadly adapted to study other fusion transcripts in resource-limited settings. Furthermore, the combination of RNase R resistance assays and divergent/convergent primer validation provided rigorous confirmation of F-circKA’s circular topology, addressing a common challenge in circRNA research.

The ethnic specificity of KANSARL, previously documented in prostate cancer (22), raises intriguing questions about its role in lung cancer disparities. While our study focused on cell lines, future investigations should explore KANSARL prevalence in diverse patient cohorts to assess its utility as a racial/ethnic biomarker. Additionally, the F-circKA/miR-6860/chKANSARL axis presents multiple therapeutic targets. Antisense oligonucleotides targeting F-circKA or miR-6860 mimics could disrupt this oncogenic loop, while chKANSARL-specific inhibitors might counteract its nuclear functions. Notably, the cytoplasmic abundance of F-circKA enhances its druggability, as extracellular vesicles enriched with F-circKA could serve as non-invasive diagnostic biomarkers.

While our findings provide compelling evidence for the KANSARL fusion axis in lung cancer, several limitations warrant attention. First, the study relied predominantly on cell line models; validation in patient-derived xenografts or primary tumors is essential to confirm clinical relevance. Second, the precise nuclear function of chKANSARL remains unclear—whether it interacts with chromatin-modifying complexes (*e.g.*, KANSL1-associated NSL histone acetyltransferase complex) or regulates specific oncogenes requires further exploration. Third, miR-6860’s broader regulatory network in lung cancer, including additional targets beyond chKANSARL, merits systematic investigation. Existing literature suggests that the KANSARL fusion is highly specific to populations of European ancestry and can be detected at high frequencies in certain cohorts. Therefore, the ethnic restriction of KANSARL necessitates population-specific studies to determine its global oncogenic impact. Finally, while our biochemical fractionation and functional data strongly support the subcellular localizations of the transcripts, direct visualization using techniques like RNA Fluorescence In Situ Hybridization could provide additional spatial resolution for F-circKA. Similarly, to further corroborate the molecular characteristics of chKANSARL and F-circKA, future studies could employ Northern blotting with junction-specific probes. This could directly confirm transcript size, discriminate potential isoforms, and assess their steady-state abundance.

In summary, our work establishes that the KANSARL fusion gene drives lung tumorigenesis through two synergistic transcriptional outputs. We demonstrate that the linear chKANSARL and circular F-circKA transcripts are not merely independent products but are functionally linked *via* a F-circKA/miR-6860/chKANSARL axis, uncovering a previously unrecognized layer of regulation within fusion gene biology. Our work underscores the importance of studying both linear and circular fusion products as integrated systems and opens new avenues for targeted therapies in fusion-driven cancers.

## Experimental procedures

### Cell lines and cell culture

The lung cancer cell lines A549 and H446, as well as the normal human bronchial epithelial cell line BEAS-2B, were obtained from the American Type Culture Collection . The normal human bronchial epithelial cell line 16HBE was obtained from the Chinese Academy of Sciences Shanghai Cell Bank. All cell lines were authenticated by Short Tandem Repeat (STR) profiling and tested negative for *mycoplasma* contamination. BEAS-2B cells were grown in bronchial epithelial basal medium (Clonetics, Basel, Switzerland). The medium was supplemented with 5% fetal bovine serum (FBS, Thermo Fisher Scientific). H446, A549, and 16 HBE cells were cultured in RPMI-1640, F-12K, and DMEM medium (HyClone), respectively, all supplemented with 10% FBS. All cells were incubated at 37 °C in a humidified incubator containing 5% CO_2_.

### Data acquisition, quality control and adapter removal

Raw RNA-seq data for normal human bronchial epithelial cells (BEAS-2B, 16HBE) and lung cancer cell lines (A427, A549, H446, H460, H1299, H2122, H2126, H2228, H23, H3122, H441, H820, H838) were downloaded from the NCBI database (https://www.ncbi.nlm.nih.gov/) with accession numbers PRJNA623863, PRJNA638541, PRJNA666088, and PRJNA673923 (40, 41, 42, 43) (Table S1). The Genome Reference Consortium Human Build 38 (GRCh38) reference genome was downloaded from Ensembl (http://ftp.ensembl.org/pub/release-107/gtf/homo_sapiens/). FastQC v0.11 (44) was used to assess the quality of the RNA-seq reads and Trimmomatic v0.39 (45) was used to filter out adapter regions. For both FastQC and Trimmomatic, default parameters were employed to assess sequencing data quality and perform adapter trimming. The processed files were used for subsequent analysis.

### Prediction of chRNAs

We used STAR (Spliced Transcripts Alignment to a Reference; https://github.com/alexdobin/STAR) software to align the preprocessed RNA-seq files, using the human reference genome data (GRCh38) as a reference. The result files generated by STAR were then analyzed by STAR-Fusion software (46) to predict possible fusion transcripts. DEEPrior (47), a deep learning tool, was used to analyze the results from STAR-Fusion to predict the potential carcinogenic fusion of linear transcripts. STAR-Fusion (v1.10.1) employed two-pass alignment (--twopassMode Basic) with stringent chimeric read detection parameters (--chimSegmentMin 12, --chimJunctionOverhangMin 8, --chimMultimapNmax 20), splice-aware mapping (--outSAMstrandField intronMotif, --alignSJDBoverhangMin 10), and paired-end overlap requirements (--peOverlapNbasesMin 12, --peOverlapMMp 0.1), alongside default settings for remaining flags. For DEEPrior, analyses were conducted using default parameters. The detection rate indicates the proportion of lung cancer cell lines in which a specific chRNA is found relative to the total number of lung cancer cell lines. The potential lung cancer-specific chimeric RNA KANSL1-ARL17A, with the highest detection rate, was screened using the "ComplexUpset" package in R (a tool for visualizing intersections in complex datasets) (48, 49). All custom scripts developed for this study have been deposited into a publicly accessible GitHub repository (https://github.com/guanxinc/chRNA-Identification).

### Genomic DNA extraction and bisection nested-PCR

Genomic DNA was isolated from cultured cells using the Takara MiniBEST Universal Genomic DNA Extraction Kit Ver.5.0. Approximately 1 × 10^6^ cells were pelleted, resuspended in PBS, and lysed with Buffer GB supplemented with Proteinase K and RNase A at 56 °C for 10 min. Ethanol was added to promote DNA binding, and the mixture was loaded onto a spin column. Contaminants and impurities were effectively removed through two-step washing using Buffer WA and ethanol-supplemented Buffer WB. Genomic DNA was eluted with 50 to 100 μl of preheated Elution Buffer after a 5-min incubation. The optimized purification steps efficiently eliminate proteins and RNA, yielding high-purity DNA suitable for sensitive downstream applications such as PCR and sequencing.

To verify the genomic origin of the KANSL1-ARL17A fusion gene, we established a “Bisection Nested-PCR”method. This approach enables rapid and accurate confirmation of the genomic source of chimeric RNAs by progressively narrowing the PCR amplification region toward the predicted fusion junction. Briefly, the first-round PCR was conducted using primers located at the exon boundaries of the parent genes (KANSL1 and ARL17A) to amplify the intronic region encompassing the putative fusion site. The PCR product obtained from this step was used as the template for subsequent rounds of PCR, with each round employing primers positioned progressively closer to the predicted genomic breakpoint. In the second round, primers were designed between the first primer pair and the fusion site to produce a shorter amplicon; the third round used primers positioned even closer to the junction, yielding a smaller fragment suitable for Sanger sequencing.

### RNA extraction and reverse transcription PCR (RT-PCR) assays

Total RNA was extracted from cells using TRIzol reagent (Invitrogen) following the manufacturer’s instructions. RNA concentration was measured using a Multiskan Spectrum microplate reader (BioTek). cDNA was synthesized from total RNA and amplified using the SuperScript IV One-Step RT-PCR System (Invitrogen, Life Technologies) and the GoScript Reverse Transcription System Kit (Promega). The RT-PCR cycling conditions were: 95 °C for 2 min, followed by 40 cycles of 95 °C for 15 s, 60 °C for 1 min, and 72 °C for 1 min. The primers used in this study are detailed in Table S2.

### Gel electrophoresis, purification, and sanger sequencing

Each PCR product (10 μl), supplemented with 2 μl of Super DNA loading buffer, was subjected to gel electrophoresis. The agarose gel concentration, running time, and voltage were adjusted according to the lengths of the DNA fragments. A 50 bp DNA marker ladder (10 μl) was loaded on both sides of each sample. After electrophoresis, the agarose gels were visualized and photographed using a GelView 6000Pro instrument (Guangzhou Biolight Biotechnology Co., Ltd). The agarose gel segments containing the PCR products were excised under ultraviolet light and purified using the E.Z.N.A. Gel Extraction Kit (Omega). The purified PCR products were dissolved in sterile water and incubated at 50 °C. The concentration of the purified products was measured using a Multiskan Spectrum microplate reader (BioTek). Qualified samples were sent for Sanger sequencing (Sangon Biotech). The primers used in this study are detailed in Table S2.

### qRT-PCR and miRNA qRT-PCR assays

qRT-PCR was used to quantify the expression levels of specific cDNAs and miRNAs. For cDNA quantification, qRT-PCR was conducted using a QuantStudio 3 Real-Time PCR System (Thermo Fisher Scientific), with the following cycling conditions: 95 °C for 2 min, followed by 40 cycles of 95 °C for 15 s and 60 °C for 1 min. GAPDH was used as the reference gene. For miRNA quantification, cDNA was synthesized from miRNA using the Bulge-Loop miRNA qRT-PCR Starter Kit (Ribobio) according to the manufacturer’s instructions. miRNA qPCR was performed on the Applied Biosystems 7500 Fast Real-Time PCR System using the miRNA qPCR Detection Kit and detected by the SYBR Green dye method. The cycling parameters for miRNA qRT-PCR were: 95 °C for 10 min, followed by 40 cycles of 95 °C for 15 s and 60 °C for 1 min. U6 snRNA was used as a reference for miRNA data normalization. Relative gene expression levels were determined by the 2ˆ-ΔΔCt method for both cDNA and miRNA analyses. The primers used in this study are detailed in Table S2.

### Cell viability assays

Cell proliferation was measured using Cell Counting Kit-8 (CCK-8, Dojindo Laboratories, Kumamoto, JPN) following the manufacturer’s instructions. Briefly, cells were seeded into 96-well plates at approximately 1500 cells per well. They were incubated for 0, 24, 48, or 72 h. After incubation, the cells were washed with PBS. Then, 10 μl of the CCK-8 reagent was added to each well, and the cells were incubated for another hour at 37 °C. The absorbance at 450 nm was measured at various time points using a Multiskan Spectrum microplate reader (BioTek).

### Plate colony formation assays

Cells were harvested and seeded in 6-well plates at 100 cells per well. They were maintained in culture medium containing 10% FBS, which was replaced every 3 days. After 14 days, the cells were fixed with paraformaldehyde and stained with crystal violet. The number of stained colonies was then counted.

### Wound-healing assays

Cells were inoculated in six-well plates at a density of 1 × 10ˆ6 cells per well, in duplicate. When the cells reached 90% confluence, a wound was created by scoring the bottom of the six-well plate with a 20 to 200 μl pipette tip. The cells were then washed with PBS and incubated at 37 °C and 5% CO_2_ for 24 h. Images were captured at 0 and 24 h after wounding using an inverted microscope (Nikon).

### Transwell invasion assays

Transwell pore polycarbonate membrane inserts were used for the invasion experiments. Cells cultured in serum-free medium with 10 g/L bovine serum albumin (MP Bio) were added to the upper chambers, which were covered with a Matrigel mixture (Corning). The lower chambers were filled with complete medium, with three replicate wells per group. After incubation for 24 h at 37 °C, the invading cells were fixed with 4% paraformaldehyde for 20 min, stained with crystal violet for 20 min, and washed with PBS to remove non-invasive cells. The cells were then photographed and counted using a microscope (Nikon).

### Lentivirus vector and transfection

The chKANSARL and F-circKA sequences were cloned into pMSCV and GV689 vectors, respectively, using specific restriction sites (BamHI/BgII and XhoI for chKANSARL; BamHI and AgeI for F-circKA) (GenePharma). The sequences are provided in Table S3.

The BEAS-2B cell lines were infected with lentiviruses containing either the human chKANSARL or F-circKA sequences to create stably transfected cell lines. Additionally, the H446 cell lines were infected with a lentivirus containing the F-circKA sequence. An empty vector was used as a negative control in all transfections. The transfected cells were selected and maintained in puromycin at a concentration of 4 μg/ml (Beyotime).

### Western blot

Total proteins were extracted from the cells using radioimmunoprecipitation buffer (Beyotime) on ice for 30 min. The cells were then centrifuged at 14,000 rpm for 20 min at 4 °C. Protein concentrations were measured using a bicinchoninic acid protein assay kit (Beyotime). Total proteins were separated using sodium dodecyl sulfate–polyacrylamide gel electrophoresis and transferred to polyvinylidene fluoride membranes (Millipore). The membranes were blocked with buffer containing 5% non-fat milk and 0.1% Tween-20. They were then incubated with a primary anti-flag antibody (Abcam, Code No. ab205606) overnight at 4 °C. An anti-β-actin antibody (Abcam) was used as a protein loading control. The membranes were then incubated with secondary antibodies IRDye 800CW goat anti-rabbit IgG and IRDye 800CW goat anti-mouse IgG (LI-COR) for 1 h at room temperature in the dark. After washing in Tris-buffered saline with 0.1% Tween-20, the labeled protein bands were visualized using an LI-COR Odyssey Infrared Imaging System.

### RNA interference

siRNA was used to knockdown chKANSARL and F-circKA. The double-stranded custom siRNA molecules were synthesized by Ribobio. Cells were seeded in 6-well plates and siRNA transfection was performed once the cells reached 50 to 60% confluency. siRNA transfection was carried out using Lipofectamine 2000 Reagent (Thermo Fisher Scientific) according to the manufacturer's protocol. Total RNA was extracted 48 h after transfection. The efficiency of siRNA-based interference was determined by qRT-PCR and western blotting. The target sequences of siRNAs are listed in Table S4.

### Tumorigenicity assays

NCG mice (Strain NO. T001475; age, 3–4 weeks; body weight, 20–25 g) were purchased from GemPharmatech. All experimental procedures involving animals were in accordance with the Guide for the Care and Use of Laboratory Animals and the institutional ethical guidelines for experiments involving animals (Ethical approval number Approval No. GY2024-036). Logarithmic growth phase H446 cells with normal expression and H446 cells with overexpressed chKANSARL were collected for inoculation. To examine the effects of chKANSARL, mice were randomly assigned to two groups: control and overexpression groups. Each group consisted of 8 mice (n = 8), with 4 males and 4 females in each group. Briefly, 2.0 × 10^6^ cells were suspended in 200 μl of a 1:1 mixture of Matrigel Basement Membrane Matrix High Concentration and complete medium. The cell suspension was then injected into the left anterior axilla of each mouse. The length (a) and width (b) of each xenograft tumor were measured every 3 days with a vernier caliper. Tumor volumes (mm^3^) were calculated using the following formula: volume = (ab^2^)/2. Mice were sacrificed 8 weeks after inoculation by cervical dislocation. The tumors were then excised, weighed, and subjected to both H&E staining and an immunohistochemical (IHC) analysis.

### Hematoxylin–eosin staining and immunohistochemical (IHC) analysis

Histological sections of the tumor xenografts were excised and fixed in 4% paraformaldehyde for 24 h. The tissues were then embedded in paraffin. Subsequently, 5-μm tissue sections were used for the diagnostic examination of tumor pathology. First, the paraffinized sections were dewaxed by sequential washing with xylene, absolute ethyl alcohol, 75% alcohol, and distilled water. H&E staining was performed on some sections to visualize morphological alterations. Other dewaxed sections were placed in a microwave oven for antigen retrieval and then blocked with BSA. After examination under a microscope, the sections were incubated with a primary antibody specific for Ki-67 (dilution, 1:100, Abcam) overnight at 4 °C. The sections were then incubated with a secondary antibody labeled with horseradish peroxidase (Beyotime) for 1 h at 37 °C. The DAB Horseradish Peroxidase Color Development Kit (Beyotime) and TMB chromogenic substrate were used to enable the peroxidase-catalyzed final brown coloration of the labeled areas. For semi-quantitative analysis, images of the stained sections were analyzed using the IHC Profiler plugin in ImageJ. The plugin categorizes staining intensity into four levels: Negative, Low Positive, Positive, and High Positive. Each category was assigned a score of 0, 1, 2, or 3, respectively. For each image, the proportion of the area in each category was calculated, and the sum of the weighted scores was defined as the IHC Score. Statistical analyses were performed based on these IHC Scores.

### Dual-luciferase reporter assays

A Dual-Luciferase Reporter Assay System kit (Promega Corp.) was used to detect the labeled reporter genes. Lipofectamine 2000 Reagent (Invitrogen) was used to cotransfect cells with F-circKA WT or mutant vectors along with a miR-6860 mimic, a miR-6860 inhibitor, and a negative control (NC) mimic (Ribobio). The F-circKA mutant sequence is provided in Table S5.

The cells were cultured in an incubator for 48 h at 37 °C. Passive lysis buffer was then used to lyse the cells. The supernatant was collected, and 20 μl were added into each well of a 96-well plate containing Luciferase Assay Reagent II (Promega Corp). The absorbance was measured at 580 nm. Next, an equal amount of Stop & Glo Reagent (Promega Corp) was added. The absorbance was measured at 460 nm, and the relative luciferase activity per well was calculated according to the manufacturer’s protocol.

### miRNA sequencing and data analysis

The total RNA concentration and purity were determined using the Nanodrop ND-1000 spectrophotometer (Thermo Fisher Scientific). RNA integrity was assessed through agarose gel electrophoresis. Sequencing was conducted with technical support from BmK using the Illumina NovaSeq6000 according to the manufacturer's protocol. miRNA differential expression calculation and analysis were performed using the DESeq2 R software package (version 1.10.1; https://bioconductor.org/packages/DESeq2/). Differentially expressed miRNAs were identified, and relevant pathway analysis was carried out.

### Pathway enrichment and statistical analysis

Gene Ontology and KEGG enrichment analyses were performed using R software (version 4.1.1; https://www.r-project.org/). The package “org.Hs.*e.g.*db” was used to identify target genes, and the “clusterProfiler” package was used to perform enrichment analysis using the Gene Ontology and KEGG databases. Statistical analyses were carried out using Prism software (version 8.0.2, GraphPad Software Inc.; https://www.graphpad.com/). Differences between groups were analyzed by Student’s *t* test or one-way analysis of variance followed by Dunnett’s *t* test. Statistical significance was considered as *p* < 0.05, and the results are presented as the mean ± standard deviation.

## Data availability

All data supporting the findings of this study are available within the article and its Supplementary Information files. Additional raw data and materials are available from the corresponding author upon reasonable request.

## Supporting information

This article contains supporting information.

## Conflict of interest

The authors declare that they have no conflicts of interest with the contents of this article.
