# Supplementary material for: The KANSL1-ARL17A fusion gene generates oncogenic chKANSARL and F-circKA RNAs that synergistically drive lung cancer progression via a novel F-circKA/miR-6860/chKANSARL axis
Source: J Biol Chem. 2026 Jan 20;302(3):111170. doi: 10.1016/j.jbc.2026.111170 (PMC12906165; doi:10.1016/j.jbc.2026.111170)
Supplement: Supplemental material [file mmc1.docx]

**Supplemental Material**

**The KANSL1-ARL17A Fusion Gene Generates Oncogenic chKANSARL and F-circKA RNAs that Synergistically Drive Lung Cancer Progression via a Novel F-circKA/miR-6860/chKANSARL Axis**


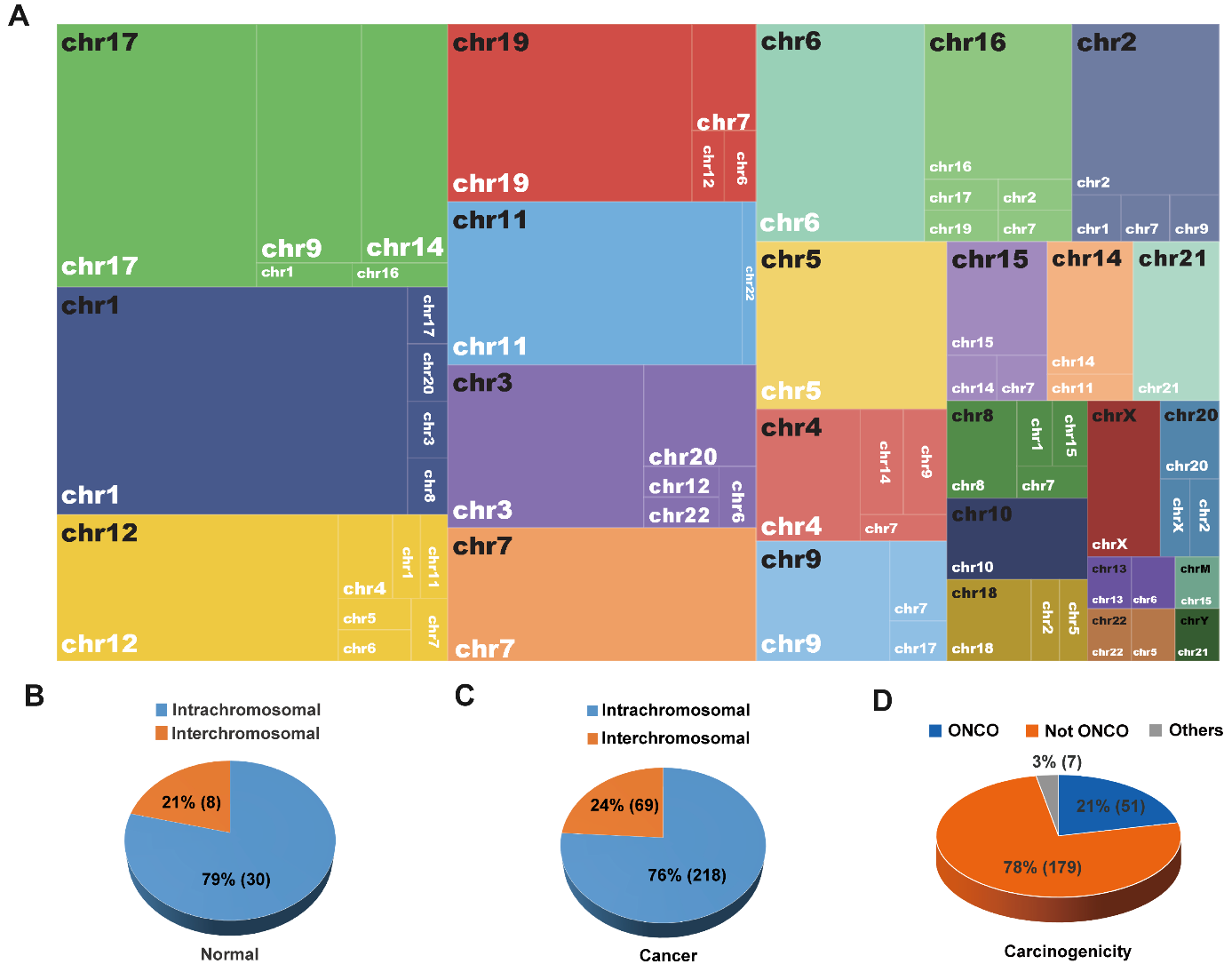
 **Fig. S1 Bioinformatic characterization of chimeric RNAs.** (A) Treemap of 237 chRNAs distributions. (B) The proportion of intrachromosomal and interchromosomal distribution of chRNAs formation pattern predicted by human lung normal epithelial cell line. (C) The proportion of intrachromosomal and interchromosomal distribution of chRNAs formation pattern predicted by human lung cancer cell line. (D) Prediction and analysis of carcinogenicity of chRNAs.


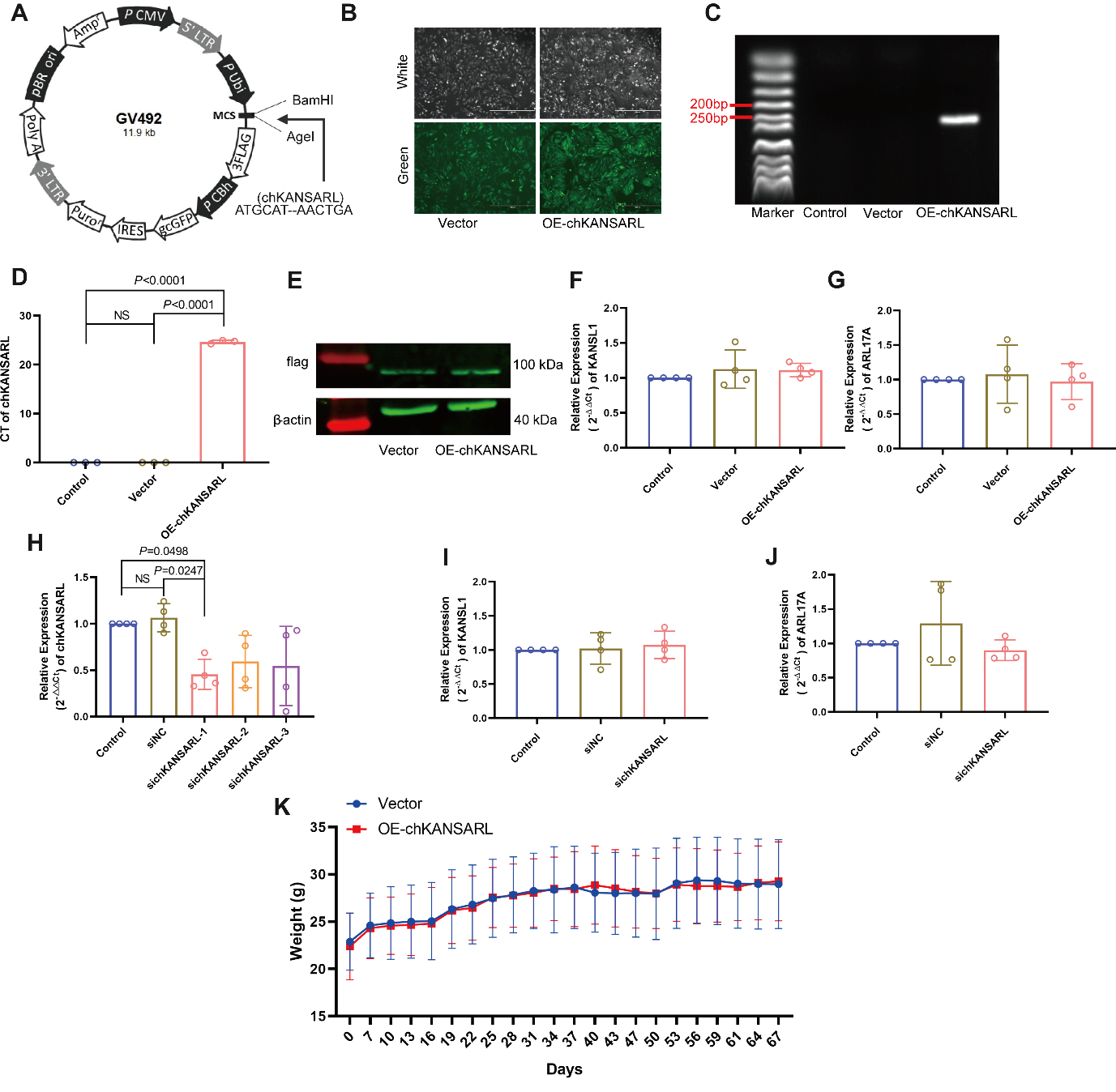


**Fig. S2 Validation of chKANSARL overexpression and knockdown systems.** (A) Schematic diagram of the chKANSARL lentiviral vector. (B) Fluorescence microscopy of lentivirus transfection in BEAS-2B cells (Scale bar = 400 μm). (C) Agarose gel electrophoresis showing the lentivirus transfection effect. (D) CT values of chKANSARL in BEAS-2B cells overexpressing chKANSARL. (E) Western blot analysis of chKANSARL overexpression. qRT-PCR analysis of (F) KANSL1 and (G) ARL17A mRNA expression in BEAS-2B cells overexpressing chKANSARL. qRT-PCR analysis of (H) chKANSARL, (I) KANSL1, and (J) ARL17A mRNA expression in H446 cells transfected with chKANSARL siRNA and negative control (NC) RNA. (K) Mouse body weight changes. All data are presented as mean ± SD from n=3 or 4 independent experiments, with individual data points plotted. Statistical significance determined by One-way ANOVA. P-values are indicated on the graph. NS, not significant.


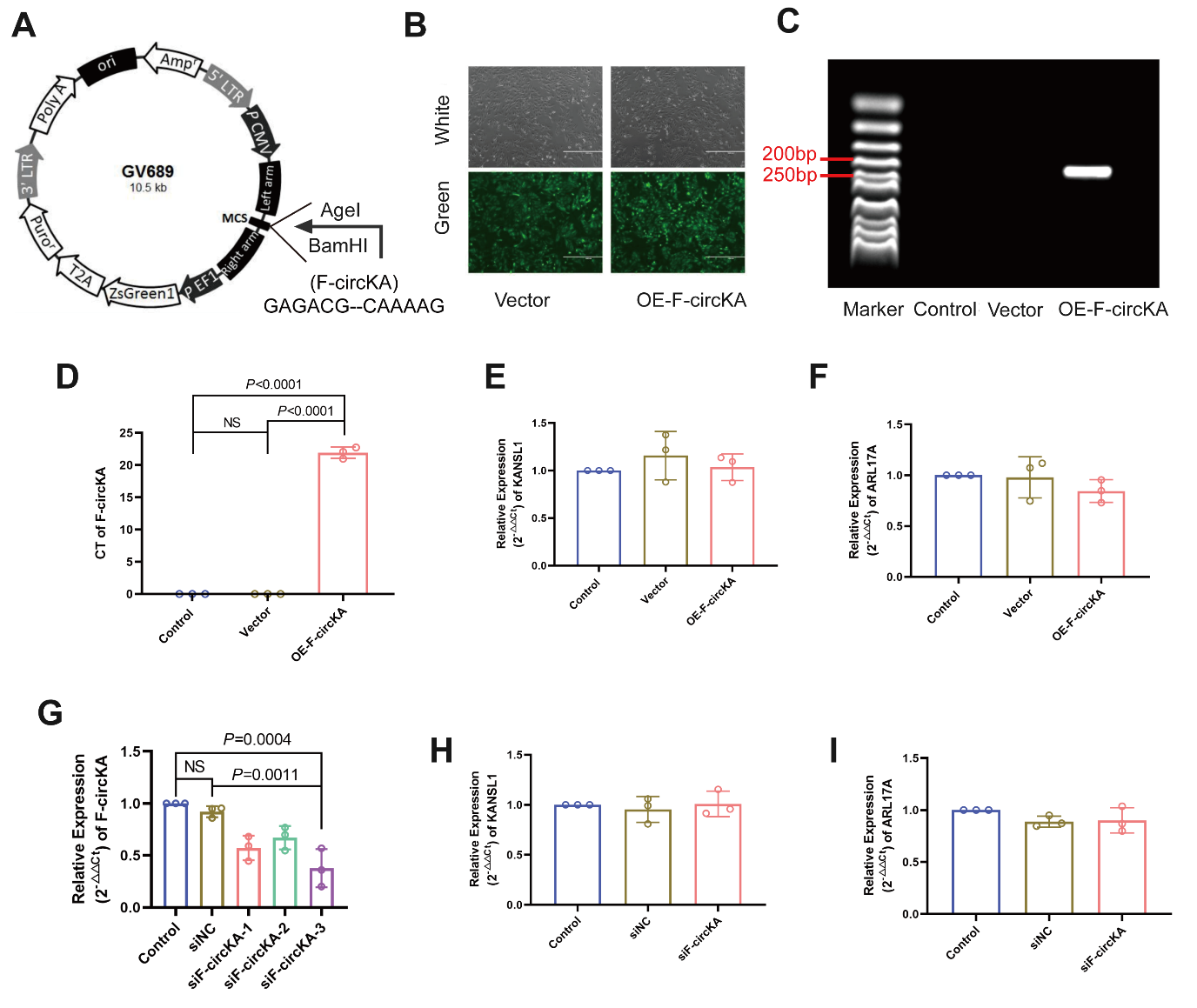


**Fig. S3 Validation of F-circKA overexpression and knockdown systems.** (A) Schematic diagram of the F-circKA lentiviral vector. (B) Observation of lentiviral transfection effect by fluorescence microscope (Scale bar = 400 μm). (C) Agarose gel electrophoresis of lentiviral transfection effect. (D) The CT values of F-circKA in the BEAS-2B cell line overexpressing F-circKA. qRT-PCR analyses of (E) KANSL1 and (F) ARL17A mRNA expression in BEAS-2B cells overexpressing F-circKA. qRT-PCR analyses of (G) F-circKA, (H) KANSL1, and (I) ARL17A mRNA expression in H446 cells transfected with siF-circKA and negative control siRNA (siNC). All data are presented as mean ± SD from n=3 independent experiments, with individual data points plotted. P-values determined by One-way ANOVA are indicated. NS, not significant.


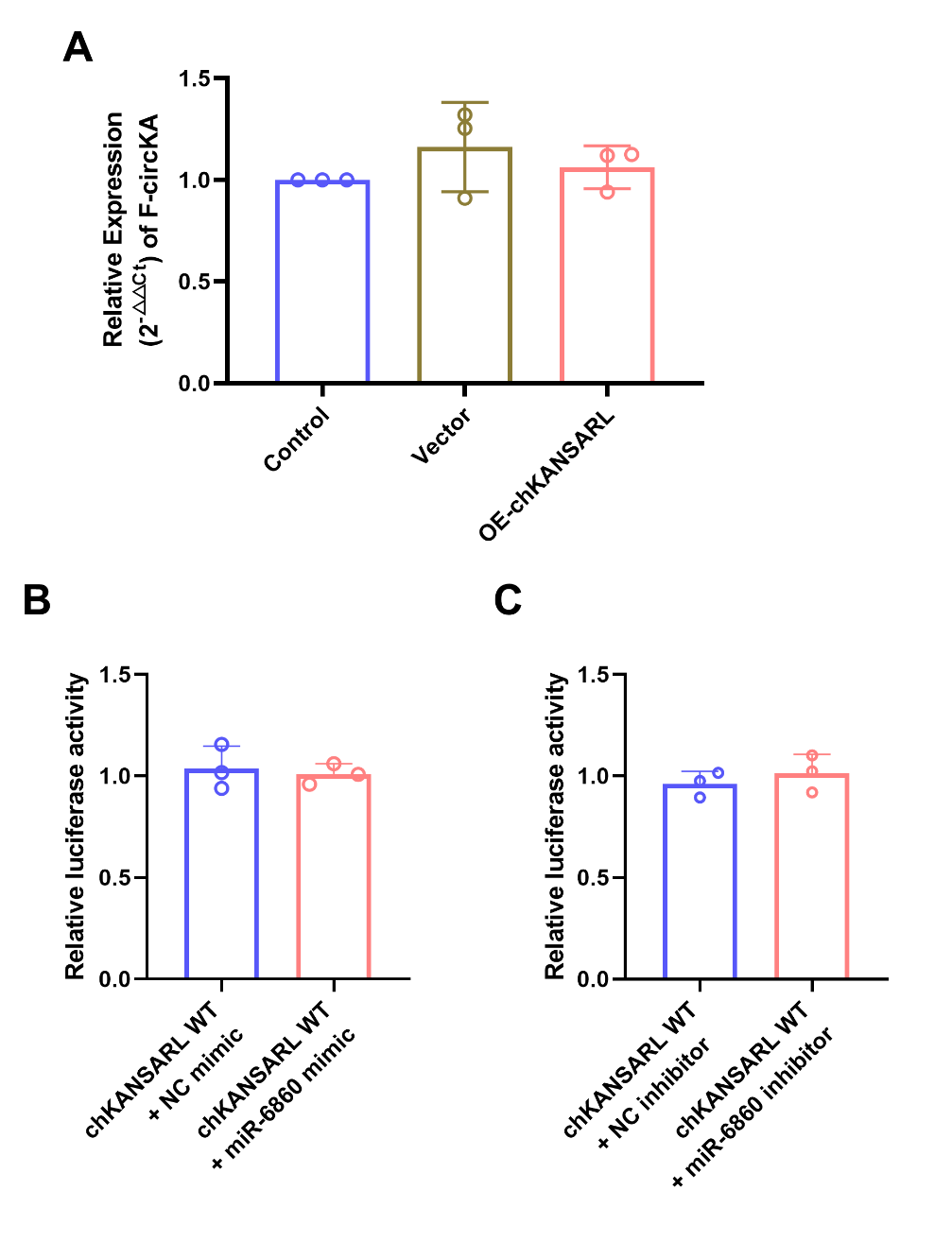


**Fig. S4 Specificity validation of the regulatory relationship between chKANSARL, F-circKA, and miR-6860.** (A) qRT-PCR analysis of F-circKA expression in H446 cells overexpressing chKANSARL. (B) Dual-luciferase reporter assay showing relative luciferase activity in H446 cells co-transfected with the chKANSARL-WT reporter vector and miR-6860 mimic or NC mimic. (C) Dual-luciferase reporter assay showing relative luciferase activity in H446 cells co-transfected with the chKANSARL-WT reporter vector and miR-6860 inhibitor or NC inhibitor. All data are presented as mean ± SD from n=3 independent experiments, with individual data points plotted. Determined by One-way ANOVA (for A) or unpaired two-tailed Student's t-test (for B and C).

Table S1 Details of RNA-seq data.

| Run ID | Cell Lines | | MB | Accession |
| --- | --- | --- | --- | --- |
| SRR11510179 | 16HBE | 1028 | | PRJNA623863 |
| SRR11510180 | 16HBE | 804 | | PRJNA623863 |
| SRR11510181 | 16HBE | 1182 | | PRJNA623863 |
| SRR11973375 | H446 | 2554 | | [PRJNA638541](https://www.ncbi.nlm.nih.gov/bioproject/PRJNA638541) |
| SRR11973376 | H446 | 2583 | | [PRJNA638541](https://www.ncbi.nlm.nih.gov/bioproject/PRJNA638541) |
| SRR11973377 | H446 | 2952 | | [PRJNA638541](https://www.ncbi.nlm.nih.gov/bioproject/PRJNA638541) |
| SRR12727820 | H460 | 4799 | | [PRJNA666088](https://www.ncbi.nlm.nih.gov/bioproject/PRJNA666088) |
| SRR12727823 | H460 | 4414 | | [PRJNA666088](https://www.ncbi.nlm.nih.gov/bioproject/PRJNA666088) |
| SRR12727827 | H460 | 5249 | | [PRJNA666088](https://www.ncbi.nlm.nih.gov/bioproject/PRJNA666088) |
| SRR12968299 | A427 | 3265 | | [PRJNA673923](https://www.ncbi.nlm.nih.gov/bioproject/PRJNA673923) |
| SRR12968300 | A427 | 3269 | | [PRJNA673923](https://www.ncbi.nlm.nih.gov/bioproject/PRJNA673923) |
| SRR12968301 | A427 | 3466 | | [PRJNA673923](https://www.ncbi.nlm.nih.gov/bioproject/PRJNA673923) |
| SRR12968302 | A549 | 2070 | | [PRJNA673923](https://www.ncbi.nlm.nih.gov/bioproject/PRJNA673923) |
| SRR12968303 | A549 | 2695 | | [PRJNA673923](https://www.ncbi.nlm.nih.gov/bioproject/PRJNA673923) |
| SRR12968304 | A549 | 2474 | | [PRJNA673923](https://www.ncbi.nlm.nih.gov/bioproject/PRJNA673923) |
| SRR12968364 | BEAS-2B | 2216 | | [PRJNA673923](https://www.ncbi.nlm.nih.gov/bioproject/PRJNA673923) |
| SRR12968365 | BEAS-2B | 1525 | | [PRJNA673923](https://www.ncbi.nlm.nih.gov/bioproject/PRJNA673923) |
| SRR12968366 | BEAS-2B | 1776 | | [PRJNA673923](https://www.ncbi.nlm.nih.gov/bioproject/PRJNA673923) |
| SRR12968388 | H1299 | 2249 | | [PRJNA673923](https://www.ncbi.nlm.nih.gov/bioproject/PRJNA673923) |
| SRR12968389 | H1299 | 2725 | | [PRJNA673923](https://www.ncbi.nlm.nih.gov/bioproject/PRJNA673923) |
| SRR12968390 | H1299 | 2108 | | [PRJNA673923](https://www.ncbi.nlm.nih.gov/bioproject/PRJNA673923) |
| SRR12968313 | H2122 | 2682 | | [PRJNA673923](https://www.ncbi.nlm.nih.gov/bioproject/PRJNA673923) |
| SRR12968314 | H2122 | 3330 | | [PRJNA673923](https://www.ncbi.nlm.nih.gov/bioproject/PRJNA673923) |
| SRR12968315 | H2122 | 2258 | | [PRJNA673923](https://www.ncbi.nlm.nih.gov/bioproject/PRJNA673923) |
| SRR12968316 | H2126 | 2635 | | [PRJNA673923](https://www.ncbi.nlm.nih.gov/bioproject/PRJNA673923) |
| SRR12968317 | H2126 | 2514 | | [PRJNA673923](https://www.ncbi.nlm.nih.gov/bioproject/PRJNA673923) |
| SRR12968318 | H2126 | 3541 | | [PRJNA673923](https://www.ncbi.nlm.nih.gov/bioproject/PRJNA673923) |
| SRR12968319 | H2228 | 2973 | | [PRJNA673923](https://www.ncbi.nlm.nih.gov/bioproject/PRJNA673923) |
| SRR12968320 | H2228 | 2312 | | [PRJNA673923](https://www.ncbi.nlm.nih.gov/bioproject/PRJNA673923) |
| SRR12968321 | H2228 | 1866 | | [PRJNA673923](https://www.ncbi.nlm.nih.gov/bioproject/PRJNA673923) |
| SRR12968331 | H23 | 2362 | | [PRJNA673923](https://www.ncbi.nlm.nih.gov/bioproject/PRJNA673923) |
| SRR12968332 | H23 | 3212 | | [PRJNA673923](https://www.ncbi.nlm.nih.gov/bioproject/PRJNA673923) |
| SRR12968333 | H23 | 2211 | | [PRJNA673923](https://www.ncbi.nlm.nih.gov/bioproject/PRJNA673923) |
| SRR12968338 | H3122 | 2161 | | [PRJNA673923](https://www.ncbi.nlm.nih.gov/bioproject/PRJNA673923) |
| SRR12968339 | H3122 | 2181 | | [PRJNA673923](https://www.ncbi.nlm.nih.gov/bioproject/PRJNA673923) |
| SRR12968340 | H3122 | 1659 | | [PRJNA673923](https://www.ncbi.nlm.nih.gov/bioproject/PRJNA673923) |
| SRR12968341 | H441 | 2926 | | [PRJNA673923](https://www.ncbi.nlm.nih.gov/bioproject/PRJNA673923) |
| SRR12968342 | H441 | 3433 | | [PRJNA673923](https://www.ncbi.nlm.nih.gov/bioproject/PRJNA673923) |
| SRR12968343 | H441 | 2988 | | [PRJNA673923](https://www.ncbi.nlm.nih.gov/bioproject/PRJNA673923) |
| SRR12968349 | H820 | 2677 | | [PRJNA673923](https://www.ncbi.nlm.nih.gov/bioproject/PRJNA673923) |
| SRR12968350 | H820 | 2733 | | [PRJNA673923](https://www.ncbi.nlm.nih.gov/bioproject/PRJNA673923) |
| SRR12968351 | H820 | 3848 | | [PRJNA673923](https://www.ncbi.nlm.nih.gov/bioproject/PRJNA673923) |
| SRR12968352 | H838 | 2767 | | [PRJNA673923](https://www.ncbi.nlm.nih.gov/bioproject/PRJNA673923) |
| SRR12968353 | H838 | 3233 | | [PRJNA673923](https://www.ncbi.nlm.nih.gov/bioproject/PRJNA673923) |
| SRR12968354 | H838 | 2395 | | [PRJNA673923](https://www.ncbi.nlm.nih.gov/bioproject/PRJNA673923) |

Table S2 The primers used in this study.

| Primer name | Sequence（5＇-3＇） |
| --- | --- |
| chKANSARL-F  chKANSARL-R | CTGAGACGCAGGTCAGAATGGA  AAATGCTGCCACAGAGGTCT |
| KANSARL-F1  KANSARL-R1 | TGTCTCAGCAGTGTTCTCAAAAATC  GGTCACCATCTTCTAAACAAAGCAA |
| KANSARL-F2  KANSARL-R2 | ACCTGATAGAGTGATGTAAGGAAGTATG  ACCAGGAAATCTCTGAAAATGAGTAATG |
| KANSARL-F3  KANSARL-R3 | CCACAGAGGAGTCACTTAGGAGGTATT  GACACAGTGGCTCATGCCTGTAATC |
| F-circKA-F(divergent)  F-circKA-R(divergent)  F-circKA-F(convergent)  F-circKA-R(convergent) | ATGTTGGCAGCCACTTCA  GTTTGTAAATGTCTGTTTGCTGACG  GGAATGGGCTGCAGACCGG  CTTTTGTGTTCTGGAAA |
| GAPDH-F  GAPDH-R | ACAGTCAGCCGCATCTTCTT  GACTCCGACCTTCACCTTCC |
| KANSL1-F  KANSL1-R | CCTTCGCTGTCTGGGATGTT  TGAGCTCTCTCCCCTTCTCC |
| ARL17A-F  ARL17A-R | CCTTCGCTGTCTGGGATGTT  GGCTTCTGGCACCTTTTGTG |

Table S3 Sequences of chKANSARL and F-circKA cloned into vectors.

| Sequence Name | Sequence（5＇-3＇） |
| --- | --- |
| chKANSARL Sequence  F-circKA Sequence | ATGCATGTACCCCTGAGACGCAGGTCAGAATGGAAATGGGCTGCAGACCGGGCAGCTATTGTCAGCCGCTGGAACTGGCTTCAGGCTCATGTTTCTGACTTGGAATATCGAATTCGTCAGCAAACAGACATTTACAAACAGATACGTGCTAATAAGGTTTCTGTGTGGAGACAGTAGAATATAAAAATAACACCTTCGCTGTCTGGGATGTTGGCAGCCACTTCAAAATCAGACCTCTGTGGCAGCATTTTTTCCAGAACTGA  GAGACGCAGGTCAGAATGGAAATGGGCTGCAGACCGGGCAGCTATTGTCAGCCGCTGGAACTGGCTTCAGGCTCATGTTTCTGACTTGGAATATCGAATTCGTCAGCAAACAGACATTTACAAACAGATACGTGCTAATAAGGTTTCTGTGTGGAGACAGTAGAATATAAAAATAACACCTTCGCTGTCTGGGATGTTGGCAGCCACTTCAAAATCAGACCTCTGTGGCAGCATTTTTTCCAGAACACAAAAG |

Table S4 Sequences of siRNA target sequences.

| siRNA Target Name | Sequence（5＇-3＇） |
| --- | --- |
| chKANSARL  F-circKA  Negative control | ACGTGCTAATAAGGTTTCT  AAAGGAGACGCAGGTCAGA  CGTACGCGGAATACTTCGA |

Table S5 Sequences of F-circKA Mutant sequences.

| Sequence Name | Sequence（5＇-3＇） |
| --- | --- |
| F-circKA Mutant | GAGACGCAGGTCAGAATGGAAATGGGCTCGAGTGGCGGCAGCTATTGTGTCGGGGACGAAGACCCTTCAGGCTCATGTTTCTGACTTGGAATATCGAATTCGTCAGCAAACAGACATTTACAAACAGATACGTGCTAATAAGGTTTCTGTGTGGAGACAGTAGAATATAAAAATAACACCAAGCGAGTCTGGGATGTTGCGTCGGAGATCAAAAAGTCACGAGTGACGGTCGATTTTTAGGTCAACACAAAAG |

Table S6 miRNA profile predicted by miRanda.

| miRNA | Score | Free energy |
| --- | --- | --- |
| hsa-miR-10395-3p | 155 | -18.31 |
| hsa-miR-106a-5p | 143 | -18.59 |
| hsa-miR-106b-5p | 143 | -15.24 |
| hsa-miR-11181-5p | 152 | -19.03 |
| hsa-miR-1184 | 141 | -13.82 |
| hsa-miR-1257 | 142 | -14.27 |
| hsa-miR-1299 | 141 | -20.58 |
| hsa-miR-1324 | 140 | -16.21 |
| hsa-miR-1538 | 147 | -29.24 |
| hsa-miR-17-5p | 143 | -16.96 |
| hsa-miR-186-5p | 140 | -9.63 |
| hsa-miR-20b-5p | 140 | -16.31 |
| hsa-miR-22-3p | 147 | -16.89 |
| hsa-miR-224-5p | 143 | -16.66 |
| hsa-miR-2277-3p | 140 | -13.27 |
| hsa-miR-29a-5p | 142 | -10.97 |
| hsa-miR-302a-3p | 144 | -17.43 |
| hsa-miR-302d-3p | 144 | -18.01 |
| hsa-miR-3064-3p | 140 | -14.1 |
| hsa-miR-3145-3p | 143 | -12.27 |
| hsa-miR-3148 | 154 | -14.05 |
| hsa-miR-3176 | 140 | -19.52 |
| hsa-miR-3192-3p | 140 | -15.68 |
| hsa-miR-3193 | 140 | -16.99 |
| hsa-miR-3616-5p | 150 | -10.49 |
| hsa-miR-3688-3p | 150 | -18.84 |
| hsa-miR-3691-3p | 152 | -15.82 |
| hsa-miR-3692-3p | 143 | -15.09 |
| hsa-miR-3713 | 142 | -15.05 |
| hsa-miR-372-3p | 146 | -21.33 |
| hsa-miR-380-5p | 141 | -21.02 |
| hsa-miR-3940-3p | 147 | -28.61 |
| hsa-miR-3978 | 153 | -20.56 |
| hsa-miR-423-3p | 159 | -30.64 |
| hsa-miR-4254 | 145 | -23.75 |
| hsa-miR-4256 | 150 | -17.31 |
| hsa-miR-4257 | 140 | -14.84 |
| hsa-miR-4293 | 141 | -15.72 |
| hsa-miR-4421 | 140 | -13.71 |
| hsa-miR-452-5p | 150 | -15.91 |
| hsa-miR-4526 | 145 | -28.11 |
| hsa-miR-4527 | 145 | -15.51 |
| hsa-miR-4635 | 151 | -21.54 |
| hsa-miR-4645-3p | 144 | -25.68 |
| hsa-miR-4676-3p | 141 | -15.51 |
| hsa-miR-4676-5p | 145 | -22.11 |
| hsa-miR-4690-3p | 140 | -21.8 |
| hsa-miR-4692 | 147 | -21.8 |
| hsa-miR-4694-5p | 150 | -11.83 |
| hsa-miR-4722-3p | 140 | -21.37 |
| hsa-miR-4727-5p | 144 | -22.54 |
| hsa-miR-4786-3p | 163 | -26.79 |
| hsa-miR-4795-5p | 167 | -21.04 |
| hsa-miR-494-3p | 152 | -11.15 |
| hsa-miR-5010-3p | 141 | -8.76 |
| hsa-miR-512-3p | 146 | -28.14 |
| hsa-miR-519a-2-5p | 143 | -16.19 |
| hsa-miR-520b-5p | 143 | -16.19 |
| hsa-miR-520c-3p | 141 | -22.21 |
| hsa-miR-526b-3p | 141 | -23.12 |
| hsa-miR-5685 | 146 | -15.47 |
| hsa-miR-5702 | 149 | -16.84 |
| hsa-miR-5706 | 145 | -21.41 |
| hsa-miR-573 | 148 | -13.31 |
| hsa-miR-575 | 148 | -19.01 |
| hsa-miR-576-5p | 142 | -19.67 |
| hsa-miR-581 | 140 | -10.37 |
| hsa-miR-584-3p | 153 | -21 |
| hsa-miR-6070 | 145 | -25.75 |
| hsa-miR-628-5p | 154 | -11.78 |
| hsa-miR-647 | 148 | -21.67 |
| hsa-miR-6503-5p | 159 | -20.27 |
| hsa-miR-654-3p | 146 | -15.04 |
| hsa-miR-671-3p | 141 | -22.12 |
| hsa-miR-6801-5p | 140 | -19.84 |
| hsa-miR-6817-5p | 143 | -19.69 |
| hsa-miR-6882-5p | 149 | -23.82 |
| hsa-miR-7107-3p | 144 | -27.39 |
| hsa-miR-7156-3p | 145 | -21.1 |
| hsa-miR-7843-3p | 146 | -15.49 |
| hsa-miR-8068 | 141 | -17.51 |
| hsa-miR-8075 | 146 | -28.29 |
| hsa-miR-873-5p | 143 | -19.04 |
| hsa-miR-875-3p | 151 | -12.89 |
| hsa-miR-885-3p | 144 | -23.7 |
| hsa-miR-889-5p | 144 | -21.09 |
| hsa-miR-892c-3p | 142 | -15.54 |
| hsa-miR-93-5p | 151 | -19.89 |
| hsa-miR-9718 | 142 | -18.22 |

Table S7 miRNA profile obtained by miRNA sequencing.

| miRNA | log2FC | Regulated | Pvalue |
| --- | --- | --- | --- |
| novel_miR_122 | -4.334982 | down | 0.0047342 |
| hsa-miR-10394-5p | -1.900659 | down | 0.0092224 |
| hsa-miR-6716-3p | -4.814208 | down | 0.0128601 |
| novel_miR_77 | -1.646579 | down | 0.0133991 |
| novel_miR_180 | -6.021635 | down | 0.01398 |
| hsa-miR-411-5p | -1.549398 | down | 0.0147367 |
| hsa-miR-3928-3p | -1.521589 | down | 0.0164814 |
| hsa-miR-4443 | -2.065217 | down | 0.0174652 |
| hsa-miR-379-5p | -2.012553 | down | 0.0190314 |
| hsa-miR-4426 | -1.321734 | down | 0.0277894 |
| hsa-miR-4511 | -4.313977 | down | 0.0284924 |
| hsa-miR-7110-3p | -4.313657 | down | 0.0287644 |
| novel_miR_190 | -1.068095 | down | 0.0301655 |
| hsa-miR-127-3p | -1.621809 | down | 0.0306014 |
| novel_miR_254 | -1.249807 | down | 0.0317852 |
| novel_miR_63 | -2.621941 | down | 0.0339767 |
| hsa-miR-491-5p | -1.213739 | down | 0.0344727 |
| novel_miR_148 | -1.218665 | down | 0.0385171 |
| hsa-miR-139-3p | -0.821556 | down | 0.0418694 |
| hsa-miR-3611 | -2.870536 | down | 0.0477836 |
| novel_miR_183 | 8.1924174 | up | 2.74E-11 |
| hsa-miR-6860 | 5.2724882 | up | 2.82E-10 |
| novel_miR_151 | 6.7355109 | up | 2.82E-06 |
| hsa-miR-629-3p | 1.4777282 | up | 0.0014236 |
| hsa-miR-6842-3p | 1.3583613 | up | 0.0134138 |
| novel_miR_189 | 2.0498323 | up | 0.0148524 |
| novel_miR_72 | 1.6515208 | up | 0.0188385 |
| novel_miR_48 | 1.2577894 | up | 0.0282927 |
| hsa-miR-26a-1-3p | 1.3794921 | up | 0.0355721 |
| hsa-miR-4454 | 1.0132346 | up | 0.0438553 |
| novel_miR_203 | 4.311188 | up | 0.0446231 |
| hsa-miR-6877-5p | 1.5983875 | up | 0.0454114 |
| novel_miR_103 | 2.4580019 | up | 0.0479681 |

Table S8 partial sequencing results of known miRNAs.

| miRNA | Pvalue | log2FC |
| --- | --- | --- |
| hsa-miR-6860 | 2.82E-10 | 5.2724882 |
| hsa-miR-6716-3p | 0.0128601 | -4.8142079 |
| hsa-miR-4511 | 0.0284924 | -4.3139768 |
| hsa-miR-7110-3p | 0.0287643 | -4.313657 |
| hsa-miR-3126-5p | 0.0519731 | 4.5566962 |
| hsa-miR-4768-5p | 0.0536693 | -4.4055688 |
| hsa-miR-101-5p | 0.0700117 | -4.4090755 |
| hsa-miR-429 | 0.0771739 | 4.0838977 |
| hsa-miR-6732-3p | 0.0923136 | -3.8712122 |
| hsa-miR-494-3p | 0.1035586 | -4.5612739 |
| hsa-miR-889-3p | 0.1109353 | -4.1182143 |
| hsa-miR-6768-5p | 0.1121893 | -4.2625908 |
| hsa-miR-4755-3p | 0.1337385 | 4.0665804 |
| hsa-miR-4999-5p | 0.1446622 | 3.4997894 |
| hsa-miR-3064-3p | 0.1596414 | 3.8834525 |
| hsa-miR-6784-5p | 0.1776146 | -3.5168995 |
| hsa-miR-543 | 0.1779235 | -3.5166203 |
| hsa-miR-4660 | 0.1785187 | -3.5160848 |
| hsa-miR-2467-5p | 0.1803413 | -3.8973771 |
| hsa-miR-3074-5p | 0.1855301 | -3.7899485 |
| hsa-miR-545-5p | 0.1891613 | 3.4686003 |
| hsa-miR-653-5p | 0.1929528 | 3.7397905 |
| hsa-miR-198 | 0.193248 | 3.7396418 |
| hsa-miR-3657 | 0.2044167 | 3.6029335 |
| hsa-miR-1256 | 0.2058022 | 3.4446702 |
| hsa-miR-6759-5p | 0.2101133 | -3.5076183 |
| hsa-miR-6839-5p | 0.2418361 | 3.8638283 |
| hsa-miR-548at-5p | 0.2424734 | 3.8638629 |
| hsa-miR-382-3p | 0.2649107 | 3.7252883 |
| hsa-miR-6746-5p | 0.273109 | -3.225838 |
| hsa-miR-6883-3p | 0.2987394 | -3.4898651 |
| hsa-miR-3145-3p | 0.3001208 | -3.4894636 |
| hsa-miR-7114-3p | 0.3153624 | -3.2668269 |
| hsa-miR-190a-3p | 0.3154134 | -3.2668586 |
| hsa-miR-10526-3p | 0.3154787 | -3.2668992 |
| hsa-miR-410-3p | 0.3158935 | -3.2671564 |
| hsa-miR-323b-3p | 0.3169949 | -3.2342704 |
| hsa-miR-3130-3p | 0.3202245 | 3.1977249 |
| hsa-miR-219b-3p | 0.3209895 | 3.1981472 |
| hsa-miR-203b-3p | 0.3215126 | 3.1984338 |
| hsa-miR-6827-5p | 0.3283061 | 3.4180607 |
| hsa-miR-580-5p | 0.3302848 | 3.2330096 |
| hsa-miR-6877-3p | 0.4154644 | -3.4028914 |
| hsa-miR-6799-5p | 0.4337009 | 3.308701 |
| hsa-miR-1255b-5p | 0.43419 | 3.3085048 |
| hsa-miR-6820-5p | 0.4420065 | 3.1883345 |
| hsa-miR-3184-3p | 0.4432526 | 3.1884263 |
| hsa-miR-6726-3p | 0.4480029 | 3.3344868 |
| hsa-miR-200b-5p | 0.4480234 | 3.3344944 |
